# High-Performance Fe–MoS_2_ Electrocatalyst for Efficient Nitrate Reduction to Ammonia: Synergistic Design for Sustainable Ammonia Production

**DOI:** 10.1021/acssuschemeng.5c04274

**Published:** 2025-10-22

**Authors:** N. Marino, R. G. Milazzo, R. Farina, C. Bongiorno, G. Fisicaro, S. Libertino, S. A. Lombardo, S. M. S. Privitera

**Affiliations:** † Institute for Microelectronic and Microsystems (IMM), National Research Council (CNR), Zona Industriale VIII Strada 5, Catania 95121, Italy; ‡ Department of Chemical Sciences, University of Catania, Viale Andrea Doria, 6, Catania 95125, Italy

**Keywords:** MoS_2_ nanosheets, electrochemical nitrate reduction NO_3_RR, electro and electroless deposition, NH_3_ electrosynthesis, bimetallic catalysts, theoretical computations

## Abstract

The electrochemical reduction of nitrate (NO_3_^–^) to ammonia (NH_3_) offers a promising route for both environmental remediation and sustainable ammonia production, yet current catalysts are limited by low efficiency and selectivity. Inspired by the active sites of nitrate reductase enzymes, this study reports the development of a high-performance electrocatalyst based on iron atoms supported on molybdenum disulfide nanosheets (Fe–MoS_2_), deposited on carbon felt. The Fe–MoS_2_ catalyst demonstrates a synergistic effect, achieving superior selectivity and productivity for NH_3_ synthesis from nitrate under neutral pH and realistic wastewater conditions (500 ppm of NO_3_^–^). Electrochemical testing reveals a maximal Faradaic efficiency of 80% at – 0.53 V versus the reversible hydrogen electrode (RHE) and a peak NH_3_ yield rate of 411 μg h^–1^ cm^–2^ at – 0.98 V versus RHE. The straightforward synthesis of the catalyst via a two-step electrodeposition and drop-casting technique ensures scalability and cost-effectiveness, addressing practical implementation challenges. Comparative studies with single-component catalysts (MoS_2_ or Fe alone) confirm the enhanced performance of the Fe–MoS_2_ system. Structural and electrochemical characterizations supported by atomistic simulations elucidate the morphology of the catalyst and active sites, while intermediate analysis provides insight into the reaction pathway.

## Graphical abstract

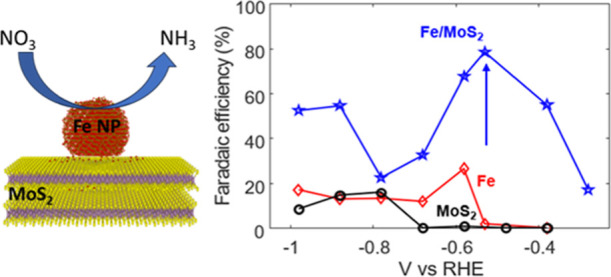

## 1 Introduction

Ammonia (NH_3_) plays a central role in several economic sectors and industrial processes, contributing significantly to the global economy and sustaining key industries.^1^ Its importance derives from its versatility as a raw material, intermediate, and final product in different applications. Indeed, in agriculture and fertilizer production, ammonia is a primary component in nitrogen-based fertilizers,^2^ promotes plant growth, and increases agricultural productivity,^3,4^ thus assuming a vital role in global food security.^5^ Moreover, ammonia has raised attention as a potential energy carrier and renewable fuel due to its high hydrogen content and ability to release it through catalytic processes.^6–9^ Recently, ammonia has been explored as a feedstock for hydrogen production through processes like ammonia cracking or direct ammonia fuel cells, offering potential solutions for energy storage and transport.^10,11^ Overall, the different roles of ammonia in agriculture field,^12^ chemical manufacturing, environmental protection, energy, and industrial processes highlights its indispensable importance in driving economic growth, innovation, and sustainability across various sectors globally.

The synthesis of ammonia stands as a cornerstone in both the industrial and agricultural sectors. Currently, the principal method for large-scale ammonia production relies on the Haber–Bosch process, contributing to substantial energy consumption and greenhouse gas emissions.^13,14^ In contrast, electrochemical synthesis of NH_3_ under ambient conditions provides a valid alternative, offering a mild and energy-efficient route, particularly through the nitrogen reduction reaction (NRR) pathway.^15–17^ However, challenges persist in electrochemical NH_3_ synthesis through NRR, primarily due to the high dissociation energy and low water solubility of nitrogen gas (N_2_), along with the competitive hydrogen evolution reaction (HER).^18^ These factors often result in modest current density and very low ammonia production, limiting the practical applicability of electrochemical NRR.^19^ An alternative solution lies in the utilization of nitrite and nitrate, ubiquitous pollutants in water systems and byproducts of various industrial processes.^20^ Nitrate pollution of soil and water—both surface and groundwater—is predominantly linked to agricultural and livestock activities, though in certain regions, it may also be associated with industrial operations. In fact, industrial nitrate waste streams with NO_3_^–^ concentration exceeding 1000 ppm can be generated during the manufacturing of explosives, fertilizers, and in the metal finishing industry.^21–23^ Excluding these high polluting industrial sectors, the admissible concentration of wastewater depends on the receiving environment: generally, 10–30 ppm for discharges into freshwater and up to 50 ppm for discharges into seawater, as defined by Directive 91/271/EEC.^24^ Converting nitrogen-containing species in wastewater into valuable ammonia not only increases their economic value but also contributes to mitigating water eutrophication.^25^ The relatively lower dissociation energy of the N=O bond (607 kJ/mol) compared to N≡N (941 kJ/mol) presents an opportunity to electrochemically reduce nitrate to ammonia at high current densities.^26^ Therefore, exploring electrochemical nitrate reduction (NO_3_RR) for NH_3_ synthesis offers a dual benefit of waste remediation and resource recovery,^27^ addressing both environmental and economic concerns simultaneously.^28^ However, the current performances of catalytic NO_3_RR are still insufficient, primarily due to the significant energy barrier of the eight-electron transfer NO_3_RR process and competition from the HER. Urgent efforts are needed to devise catalysts that can efficiently enhance the NO_3_RR while suppressing the HER.

To date, extensive exploration has been conducted on various noble metals like Pt and Pd, as well as bimetallic combinations such as Pd–Cu, for nitrate electroreduction catalysis.^29,30^ However, the practical application of these materials is hindered by their high cost and limited availability. In contrast, transition metal nanoparticles have emerged as a promising alternative for electrode materials in the electrocatalytic nitrate reduction reaction.^31^ Commonly investigated transition metals include iron (Fe), cobalt (Co), nickel (Ni), copper (Cu), and their respective alloys and compounds.^32^ These catalysts enable the conversion of nitrate ions to ammonia through multistep electrochemical reactions, including nitrate reduction to nitrite (NO_2_^–^) and/or hydroxylamine (NH_2_OH), preceding ammonia formation^33,34^ and hydrogen reduction. Effective catalyst design and optimization are therefore essential for enhancing the performance of transition metal catalysts in electrochemical nitrate reduction. One of the aspects to be taken into account is the low binding affinity and nucleophilicity of nitrate on transition metals due to its planar symmetrical resonant structure, which distributes the negative charge equally across all three oxygen atoms and reduces the electron density available at any single oxygen for coordination. This makes it challenging for NO_3_^–^ to interact effectively with metal catalysts. As a result, harsh reaction conditions, such as high temperatures, UV-light photolysis, and strongly acidic pH, are often necessary to facilitate the reduction of NO_3_^–^. These demanding conditions limit the practical application of many artificial catalysts in nitrate reduction reactions.

In contrast, nature has evolved highly efficient mechanisms for catalyzing the NO_3_RR under mild conditions. Nitrate reductase (NRase) enzymes are exemplary in this regard, serving as the most efficient NO_3_RR catalysts in neutral aqueous solutions.^35^ NRase selectively reduces NO_3_^–^ to nitrite (NO_2_^–^) at approximately 150 mV versus the normal hydrogen electrode at pH 6 and ambient temperature.^36,37^ This process occurs without the need for harsh reaction conditions, proving the efficiency of the enzyme. All known naturally occurring NRases utilize molybdenum (Mo) as the catalytic center. These enzymes feature a mononuclear Mo atom coordinated by oxo- and dithiolene sulfur ligands connected to one or two protein groups. The structural and functional active sites of natural enzymes (nitrate reductase and nitrogenase), including the FeMo cofactor and sulfur, suggest that isolated and well-coordinated Fe atoms offer significant advantages in improving the efficiency and selectivity of the electrochemical reduction of nitrate to ammonia.

Inspired by the structure of active sites of enzymes, here we have fabricated a new catalyst based on Fe supported on MoS_2_ nanosheets (Fe–MoS_2_) deposited on Carbon felt (C-felt) for the electrocatalytic NO_3_RR. The preparation method is simple and sustainable, since it adopts room-temperature processes and earth abundant materials. The designed catalyst has been tested at pH around 7 under realistic conditions for wastewater (500 ppm of nitrate), i.e., in the midrange between low environmental levels required for drinking water (10–50 ppm according to the World Health Organization^38^ and the European Directive 98/83/EC^39^) and high industrial concentrations (up to 1000 ppm). The catalyst exhibited high selectivity and productivity for NH_3_ electrosynthesis, achieving a maximal Faradaic efficiency (FE) of 80% at – 0.53 V versus the reversible hydrogen electrode (RHE) and a peak yield rate of 411 μg h^–1^ cm^–2^ at −0.98 V vs RHE. Related Fe- and MoS_2_-based catalysts have been recently reported.^40^ Here, we explicitly differ by employing a room-temperature, scalable synthesis, testing at neutral pH at realistic NO_3_^–^ concentrations (500 ppm) and providing atomistic ML-based simulations that elucidate the observed synergy. The results have been compared with those obtained by using separately iron oxide or MoS_2_, demonstrating enhanced performance due to the synergistic effect of the Fe–MoS_2_ catalyst and highlighting the potential of Fe–MoS_2_ nanosheets as efficient catalysts for NH_3_ production from NO_3_^–^. Initial and final nitrate concentration and nitrite concentration were also measured to estimate the conversion rate through electroreduction to explore the potential reaction intermediates formed during the process of converting nitrate to ammonia. To explore the atomistic origin of the enhanced catalytic performances of the C-felt-MoS_2_–Fe catalyst, atomistic simulation with state-of-art machine learning interatomic potentials have been performed for the composed system.

## 2 Methods

### 2.1 Materials

Carbon felt (C-felt Freudenberg H23C2) was purchased from Freudenberg Performance Material; Trisodium citrate dihydrate (Na_3_C_6_H_5_O_7_·2H_2_O > 99.0%); Salicylic acid (C_7_H_6_O_3_ 99%); Sodium nitroferricyanide­(III) dihydrate Na_2_[Fe­(CN)_5_NO]·2H_2_O 98%); Sodium hypochlorite solution (NaClO, 11–15%); Phosphate Buffered Saline Tablets; Sodium hydroxide NaOH 97+%; Iron­(III) chloride reagent grade 97%; Molybdenum­(IV) sulfide 98% powder; Ammonia standard solution 1000 ppm as N; Potassium nitrite 99.0%; 1-Methyl-2-pyrrolidinone 99%; and 2-Propanol 70% in H_2_O were purchased from Sigma-Aldrich or Alfa Aesar and used without further purification.

Copper­(II) sulfate pentahydrate (CuSO_4_·5H_2_O), manganese­(II) chloride (MnCl_2_), and potassium chloride (KCl) were purchased from Merck KGaA (Darmstadt, Germany); screen-printed carbon electrodes (SPCEs, cod. ref 150 and ref. CL110) were purchased from Metrohm DropSens s.r.l. (Origgio, VA, Italy). Copper and Manganese electrodeposition and electrochemical measurements were performed with the Palmsens4 electrochemical workstation by PalmSens BV (C-PS4-BP.F2.10, GA Houten, The Netherlands).

### 2.2 Catalyst Preparation

Catalyst C-felt-MoS_2_–Fe was prepared in a two-step procedure: (i) electrodeposition of exfoliated MoS_2_ on C-felt, (ii) FeCl_3_ solution-based deposition through a drop-casting method on C-felt-MoS_2_. MoS_2_ exfoliation was performed by a liquid-phase probe ultrasonicator-assisted method using MoS_2_ bulk powder. The MoS_2_ nanosheets obtained from liquid exfoliation generate electron-rich, highly hydrophilic (therefore dispersible in water) and negatively charged particles; this condition is necessary for electrodeposition technique.^41^ Initially, the MoS_2_ was dispersed in *N*-methyl-2-pyrrolidone with an initial concentration of 5 mg/mL. The exfoliation process was carried out in 8 h of sonication at a frequency of 60 kHz, performed in a 20 mL glass vial with cycles of 0.8 s ON and 0.2 s OFF. After sonication, the resulting suspension was centrifuged for 1 h at 4500 rpm to separate the unexfoliated MoS_2_ sheets. The top one-third of the suspension, which contained well-exfoliated MoS_2_ nanosheets, was collected for the next step: electrophoretic deposition. This deposition process involved applying a voltage of 10 V between two electrodes immersed in 2-Propanol 70% in a H_2_O solution. A C-felt, measuring 1 × 1 cm^2^, was used as the positive electrode (substrate). To avoid uncontrollable doping of the prepared structures, instead of graphite, a 0.2 mm thick stainless steel sheet was used as the negative counter electrode. The electrodes were spaced 1 cm apart, and the deposition time was set at 60 min. After deposition, the prepared structure was dried at room temperature for 12 h to ensure complete evaporation of the solvent. This method is significant as it combines the advantages of liquid-phase exfoliation and electrophoretic deposition, providing a controlled and efficient way to produce MoS_2_ nanosheets. The detailed steps of the synthesis process are schematically illustrated in Figure 1.

**Figure 1:**
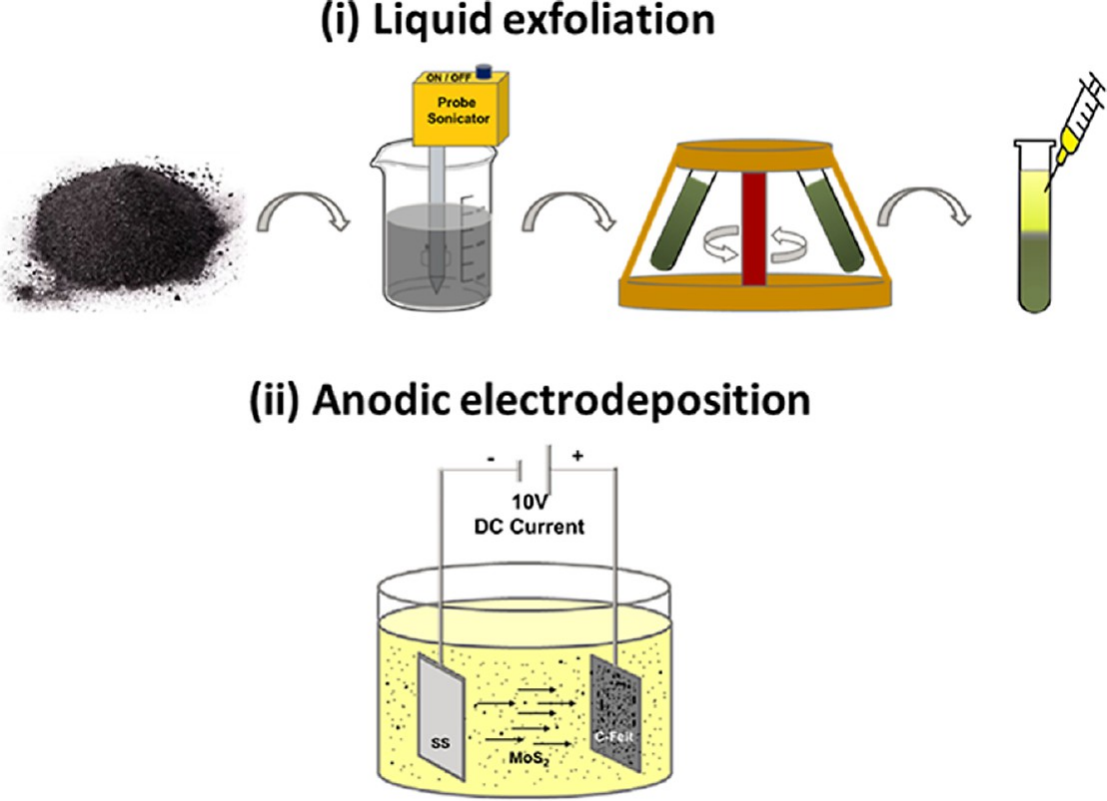
Deposition procedure for MoS_2_ nanosheets on C-felt: (i) liquid exfoliation and (ii) anodic electrodeposition.

For the iron deposition process, a 1 mM FeCl_3_ aqueous solution was prepared. Then, 0.6 mL of FeCl_3_ solution was pipetted on the C-felt substrate measuring 3 × 1 cm^2^. This substrate was positioned on a hot plate set to 80 °C to promote the clustering of a thin layer of FeCl_3_, by solvent evaporation. The substrate is then dipped in a stirred solution containing 100 mM NaBH_4_ to promote the iron reduction and chloride removal from the surface. All of the steps are schematically represented in Figure 2.

**Figure 2:**
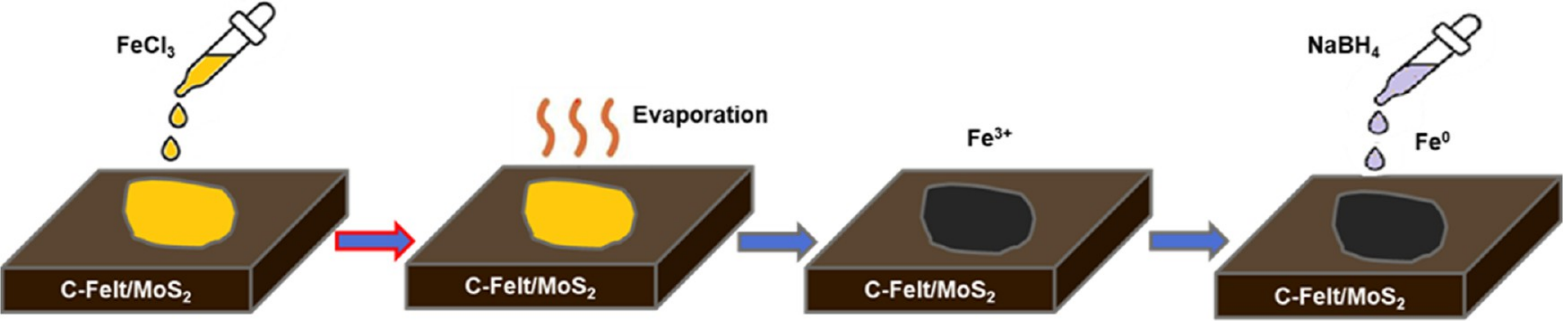
Deposition of FeNPs based on FeCl_3_ evaporation and subsequent reduction in NaBH_4_ solution.

### 2.3 Structural and Electrochemical Characterizations

To understand the structure of the deposited materials, Micro-Raman spectroscopy was carried out using a Horiba Labram HR Evolution instrument, equipped with a 532 nm laser. The deposited catalysts were analyzed by using a 100× objective and a grating at 1800 cm^–1^, to ensure high resolution.

Investigations by Scanning electron microscopy (SEM) were carried out with a Jeol JSM-IT800 SHLs microscope equipped with a windowless Gather X JEOL energy-dispersive detector (EDS).

High-Resolution Transmission Electron Microscopy (HRTEM) and Scanning TEM (STEM) have been performed by using a JEOL 2010F microscope operating at 200 keV with the aim of studying in detail the structure and morphology of the catalyst. Samples for microscopical analyses were prepared on carbon grids, following the same procedure adopted for the deposition on a C-felt. EELS spectra were performed on a probe-corrected JEOL ARM 200F equipped with a cold field emission gun operated at 200 kV.

Electrochemical experiments were carried out with an IVIUM Vertex 10A potentiostat in an aqueous solution containing 0.01 M Phosphate Buffer (pH 7) and 500 ppm of NO_3_^–^; thus, ammonia is produced as ammonium salt (NH_4_^+^). PBS has been chosen as an electrolyte in order to provide a stable, neutral-pH environment (pH ≈ 7.4) that closely mimics the conditions of real wastewater streams, where nitrate concentrations around 500 ppm are typically encountered. Its buffering capacity prevents pH drift during prolonged electrolysis and thus ensures that both the catalyst’s performance and the competing hydrogen evolution reaction can be assessed under practically relevant, mild conditions. We adopted a divided H-type electrolysis cell in combination with a classical three-electrode configuration (see Figure SI1) wherein the C-felt loaded with the catalyst served as the working electrode, a Pt-wire served as the counter electrode, and Ag/AgCl (3 M KCl) served as the reference electrode. The anodic and the cathodic compartments were separated by Agfa’s ZIRFON PERL.^42^ Potentials were converted from the Ag/AgCl scale to RHE by employing the following formula:

1 $$E_{\left(RHE\right)}=E_{Ag/AgCl}+0.059pH+E_{Ag/AgCl}^{o}$$

where *E* ^o^_Ag/AgCl_ = 0.207 V at 25 °C and *E*_Ag/AgCl_ is the working potential.

Linear Sweep Voltammetry (LSV) measurements were collected with a scan rate of 40 mV s^–1^, electrochemical impedance spectroscopy (EIS) analysis was performed from 10 kHz to 0.1 Hz with a signal amplitude of 10 mV and the corresponding Nyquist plots have been modeled assuming a typical R­(RC) equivalent circuit, and the chronoamperometry measurements (*i*–*t*) were acquired for 2400 s at different working potentials, from −0.28 V to −0.98 V, with a step of −0.1 V. All electrode potentials were iR corrected.

### 2.4 Ammonia Quantification

The quantity of NH_3_ present in the solution after the chronoamperometry experiment was quantified via colorimetry by employing the indophenol blue method. This method relies on the modified Berthelot reaction,^43^ where ammonia reacts with hypochlorite (ClO^–^) to form monochloramine (NH_2_Cl). Subsequently, monochloramine reacts with salicylic acid (C_7_H_6_O_3_) in the presence of a catalyst, typically sodium nitroprusside (Na_2_Fe­(CN)_5_NO·2H_2_O), to form a green indophenol compound. The intensity of the color is directly proportional to the ammonia concentration, allowing for visual or spectrophotometric quantification. Further details and the calibration curve obtained from UV–vis spectra of solutions with known concentrations are shown in Figure SI2. To improve the reliability of the test, we have adopted Ammonia ISE (Ion selective electrode) as an alternative measurement method. Our Ammonia ISE (9512HPBNWP) is provided by ThermoScientific and connected to 4-Star pH/ISE). Results are shown in the Supporting Information.

### 2.5 Nitrates and Nitrites Quantification

Nitrate (NO_3_^–^) and Nitrites (NO_2_^–^) concentrations in the solutions, before and after the chronoamperometric experiments, were quantified by using innovative electrochemical sensors based on screen-printed electrodes (SPEs). A screen-printed electrochemical cell consists of three electrodes: a working electrode (WE), which can be functionalized with materials selective for the target analyte; a reference electrode (RE), which ensures the precise control of the WE potential; and a counter electrode (CE), which completes the electrochemical circuit. The SPE electrochemical cell used to develop NO_3_^–^ and NO_2_^–^ sensors consists of a carbon WE, silver RE, and platinum CE.^44^ The nitrate sensor was fabricated by cyclic voltammetry electrodeposition of copper microflower crystals on carbon WE. Fabrication and performance details can be found in refs 45 and 46 . The nitrite sensor was fabricated by cyclic voltammetry electrodeposition of CuO and MnO_2_ on carbon WE. Nitrate and nitrite concentrations were determined using Linear Sweep Voltammetry (LSV) by monitoring the NO_3_^–^ reduction peak current at – 0.86 V vs Ag and the NO_2_^–^ oxidation peak current at +0.8 V vs Ag, respectively. Calibration curves obtained in 0.01 M phosphate buffer solution (PBS) are shown in Figures SI3 and SI4.

### 2.6 Computational Methodology

Atomistic simulations are a valuable tool to inspect and characterize the local morphologies of heterogeneous materials characterized by complex interfaces. To explore the interactions and the configurational space of the C-felt-MoS_2_–Fe system, molecular dynamics (MD) in the canonical ensemble *NVT* (moles *N*, volume *V*, and temperature *T* are kept constant) have been performed using the Atomic Simulation Environment (ASE) python library.^47^ In the *NVT* ensemble, the system is allowed to exchange heat with the outer space so that the temperature stays constant.

To properly model the target composed catalysts, atomistic simulations of thousands of atoms with nearly quantum accuracy are needed.^48^ The composite nanosheets hold up to five different elements, characterized by different phases and complex local arrangements. Advances in machine learning have led to the development of foundation models for atomistic materials simulations and design, enabling quantum-accurate descriptions of interatomic forces across diverse compounds at reduced computational cost.^49^ Foundation machine-learning potentials (fMLPs) are trained across nearly all chemical elements and can be combined to describe almost arbitrary compounds. To accurately model the potential energy surface of the C-felt-MoS_2_–Fe system, we rely on the MACE-MP-0 foundation machine learning potential (model large MACE_MPtrj_2022.9. model), trained on 1.6 M bulk crystals of Materials Project (MP) and covering 89 elements.^50^ Grimme D3 dispersion interactions have been included^51^ by the accelerated D3 PyTorch implementation.^52^

All structures were relaxed until forces were less than 1.0 meV/Å. C-felt exposed terminations have been modeled by crystalline graphite composed by two AB-stacked graphene layers (C_mp-48 structure from Materials Project).^53^ The two-layer carbon substrate holds a total of 3584 atoms. The optimized substrate extends for 68.24 Å × 68.94 Å on the periodic surface plane with an interlayer distance of 3.44 Å. MoS_2_ nanosheets are modeled by two layers of hexagonal MoS_2_ (MoS_2__mp-2815 structure from MP). The two-layer MoS_2_ substrate holds a total of 3432 atoms. The optimized substrate extends for 71.38 × 69.74 Å on the periodic surface plane with an interlayer distance of 6.16 Å. The predicted interlayer distance for both materials proves the reliability of the MACE-MP-0 foundation machine learning potential for our application. A stoichiometric iron oxide nanoparticle (NP) with a radius of 2.0 nm has been generated from crystalline Fe_2_O_3_ hematite (Fe2O3_mp-19770 structure from MP). The Fe_2_O_3_ nanoparticle holds 3185 atoms. Panels (0) of Figure 8 show the composed structures for C-felt/NP and MoS_2_/NP.

## 3 Results and Discussion

To gain a deeper understanding of the interaction between molybdenum disulfide and iron deposited on carbon felt and to evaluate their behavior in electrochemical experiments, we designed a study focusing on three distinct catalyst configurations: C-felt-Fe, C-felt-MoS_2_, and C-felt-MoS_2_–Fe. This investigation aims to elucidate the synergistic or independent effects of MoS_2_ and iron on the catalytic performance, particularly in the context of electrochemical nitrate reduction. The C-felt-Fe catalyst is composed solely of iron deposited on a carbon-felt substrate. This configuration allows us to evaluate the inherent catalytic properties of iron, particularly its role in facilitating electrochemical reactions. The second catalyst, C-felt-MoS_2_, involves the deposition of MoS_2_ nanosheets onto carbon felt. MoS_2_, as a well-known 2D material with unique electronic and catalytic properties, provides a baseline for understanding its effectiveness as a catalytic material as well as its Faradaic efficiency and ammonia production rates. The final catalyst, C-felt-MoS_2_–Fe, combines both materials, where iron is deposited onto a MoS_2_-modified carbon-felt substrate. This configuration is crucial for exploring the interaction between MoS_2_ and iron, particularly the potential synergy between these materials.

### 3.1 Structural Characterization

#### 3.1.1 C-Felt-Fe Catalyst

The method adopted for the deposition of iron has been investigated in a previous work.^54^ In summary, by following this solution-based approach, we performed surface coating of the C-felt fibers with a discrete distribution of iron-based nanoparticles. The morphological and structural characterizations of Fe deposited using the proposed approach are shown in Figure 3. The SEM analyses of Figure 3a,b show that iron-based nanoparticles are uniformly distributed over the C-felt and exhibit a very small size, typically in the range 10–20 nm. TEM analysis at high resolution, reported in Figure 3c, clearly indicates that the particles have a core–shell structure with a core of metallic iron and a shell of iron oxide or hydroxide. Fast Fourier Transform (FFT) of Figure 3d,e reveals lattice spacing amounting to 2.25 Å, 2.41 Å, and 2.06 Å which correspond to FeOOH (121), Fe_3_O_4_(222),^55^ and Fe(110),^56^ respectively. They were acquired in regions 1 and 2 corresponding to the core and shell, respectively, and the iron crystalline diffraction is present only in the inner region of the particle. The Raman spectrum shown in Figure 3f evidences the presence of vibrational modes that can be attributed to the alpha phase of FeOOH,^57^ consistent with the TEM observation.

**Figure 3:**
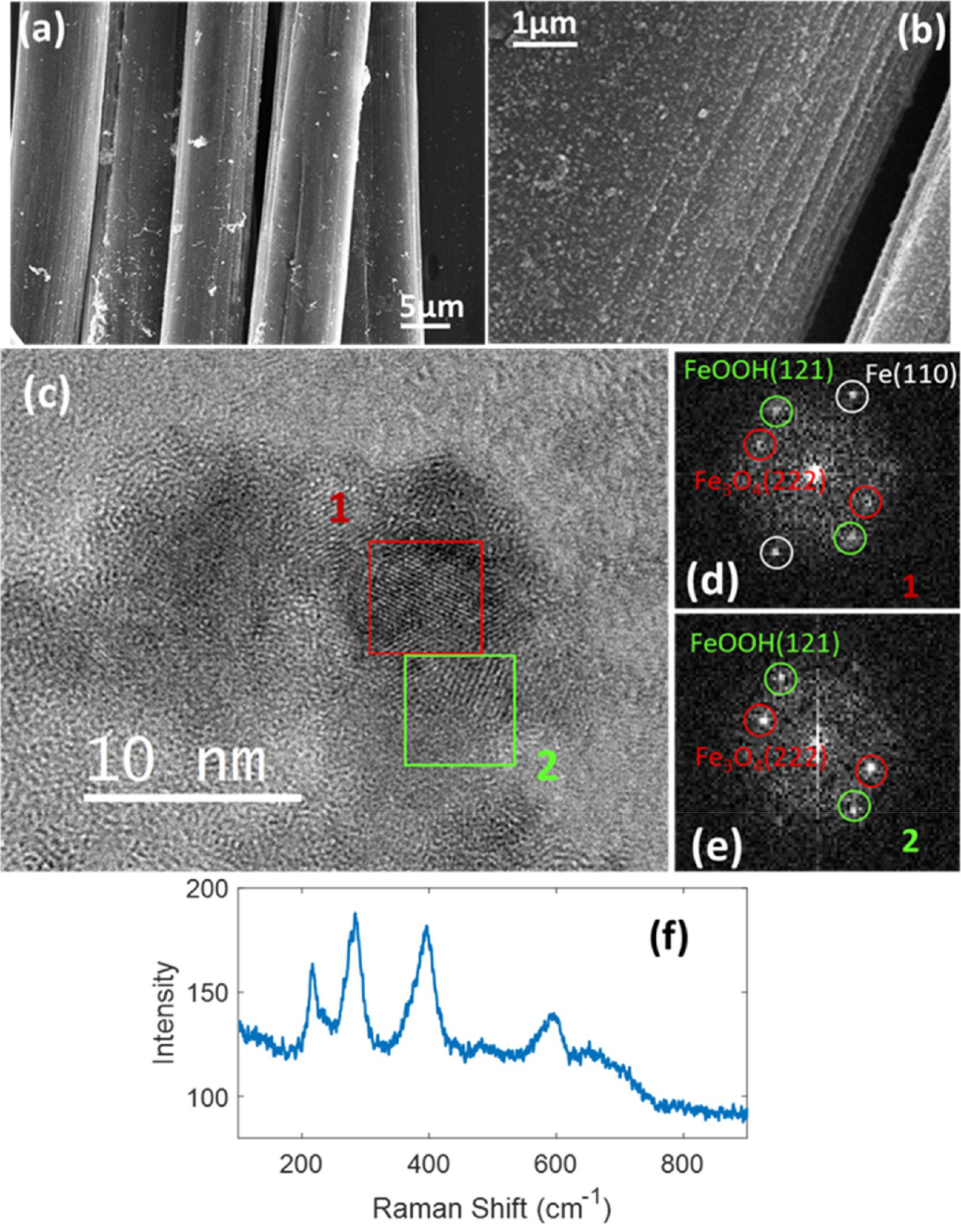
Morphological and structural analyses of the C-felt-Fe catalyst. (a,b) SEM of Fe on C-felt; (c) HRTEM of FeNPs; (d) the FFT of region 1, showing the diffraction pattern of both crystalline and oxidized iron and (e) the FFT of region 2, reporting the diffraction spots of oxidized iron only; (f) Raman spectrum of the Fe catalyst on C-felt.

#### 3.1.2 C-Felt-MoS_2_ Catalyst

MoS_2_ electrodeposited on C-felt has been investigated by SEM, as shown in Figure 4a,b at two different magnifications. The energy-dispersive spectroscopy (EDS) confirms the presence of MoS_2_ (see Figure SI5). STEM analyses reported in Figure 4c show the presence of MoS_2_ flakes, with different orientations. High-resolution TEM analyses, shown in Figure 4d,e, evidence the typical diffraction pattern of the basal plane of the 2H structure with the (200) diffraction spot, corresponding to a spacing of 2.7 Å, as shown in the FFT of Figure 4d; in the normal direction instead, we found 4–6 monolayers with an interplanar distance of 6.3 Å, as usually reported for (002) MoS_2_.^58^ The value of 6.16 Å obtained by atomistic structural optimization of MoS_2_ using the MACE-MP-0 foundation machine learning potential is also in good agreement with the experimental one.

**Figure 4:**
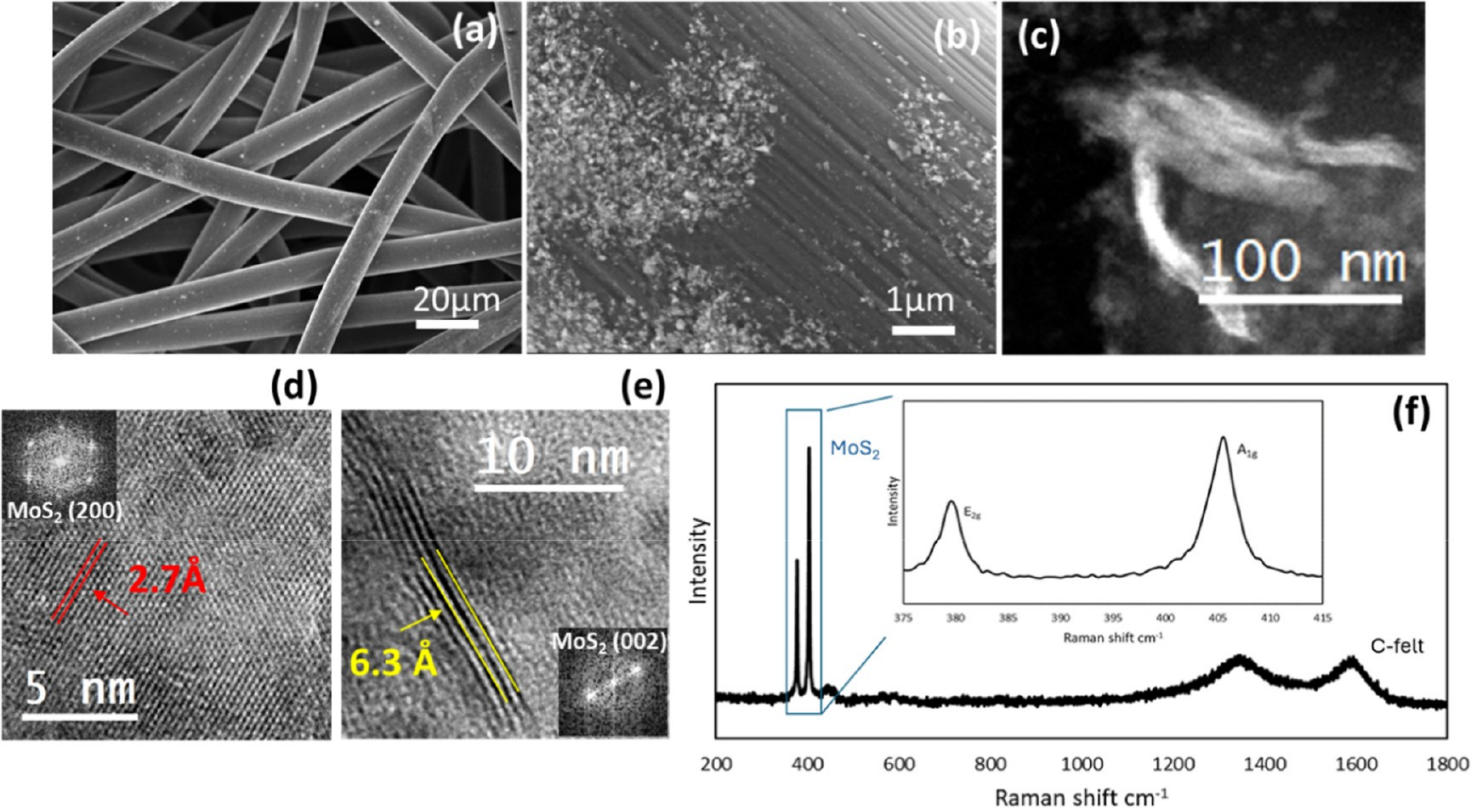
Morphological and structural analyses of the C-felt-MoS_2_ catalyst: (a) and (b) SEM micrographs, (c) STEM micrograph showing flakes randomly arranged on the substrate; (d) HRTEM of the basal plane of MoS_2_ (see the inset) with an interatomic spacing of 2.7A; (e) MoS_2_ nanoflakes along the stacking direction with an interatomic distance of 6.3 Å; (f) Raman spectrum of MoS_2_.

The Raman spectrum, plotted in Figure 4f, exhibits vibrational modes at about 382.5 cm^–1^ and 407 cm^–1^ that can be assigned to the E_2g_^1^ and A_1g_ modes of the hexagonal (2H) MoS_2_ crystal, respectively. The peaks at 1350 cm^–1^ and 1600 cm^–1^ are typical of the carbon-felt supporting electrode. The relatively large peak width (∼5 cm^–1^) and weak intensity of the E_2g_^1^ peak suggest that the crystal structure of MoS_2_ is not perfect.^59^

The intensity ratio between the E_2g_^1^ peak and the A_1g_ peak is on average around 0.7, with values ranging from 0.5 to 0.9, suggesting that the MoS_2_ flakes are oriented mainly horizontally (E_2g_^1^/A_1g_ around 0.7–0.8), although some flakes, characterized by lower values of the ratio E_2g_^1^/A_1g_ (0.4–0.5), are vertically aligned.^60^ Overall, the morphological and structural characterizations suggest that the MoS_2_–C-felt is composed of crystalline 2H MoS_2_ flakes, with 4–6 monolayers and with a high density of edges and defects.

#### 3.1.3 C-Felt-MoS_2_–Fe Catalyst

The third catalyst, C-felt-MoS_2_–Fe, combines both materials, where iron is deposited onto a MoS_2_-modified carbon-felt substrate. This configuration is crucial for exploring the interaction between MoS_2_ and iron, particularly the potential synergy between these materials.

Figure 5a,b shows the SEM images of the C-felt-MoS_2_–Fe catalyst (see Figure SI6 for EDX elemental mapping). Flower-like structures are observed, which can be attributed to either Fe oxide or MoS_2_ nanosheets or nanopetals. Further details can be obtained by TEM analysis. Figure 5c,d evidences the widespread presence of MoS_2_ flakes, with a typical spacing of 6.3 Å (002). Fe-based nanoparticles are also observed. According to the FFT of the particles, the crystalline structure of iron oxide and hydroxide can be identified (see the (222) Fe_3_O_4_ and the (121) FeOOH diffraction patterns with lattice spacings of 2.41 and 2.25 Å, respectively). In the case that the nanoparticles overlap MoS_2_, the crystalline Fe representing the core of the iron NP in the CF/Fe catalyst is no more detectable.

**Figure 5:**
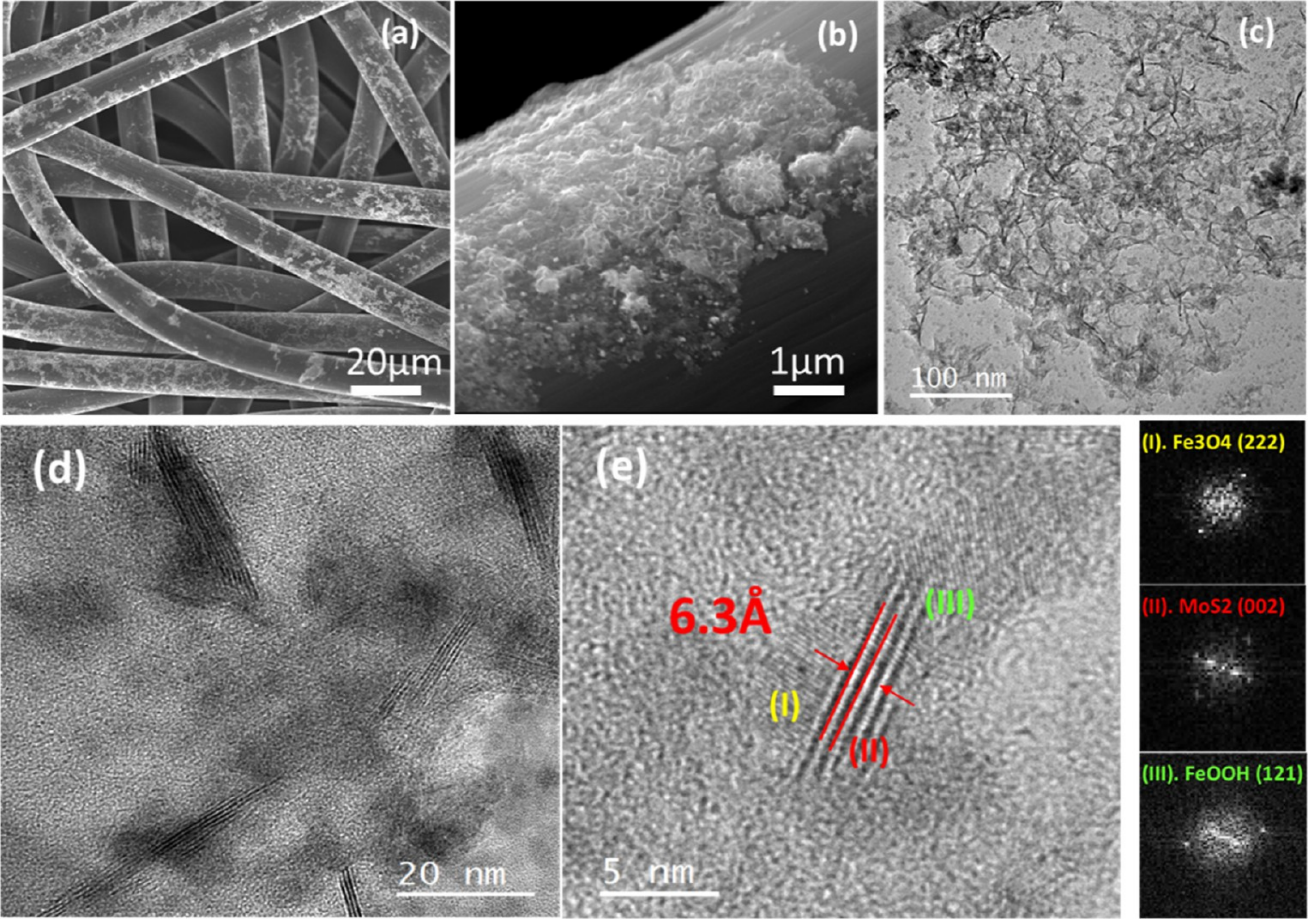
Morphological and structural analyses of the C-felt-MoS_2_–Fe catalyst: (a,b) SEM and (c) TEM bright field micrographs showing the flower-like structure of Fe oxide; (d) HRTEM micrographs of MoS_2_ nanosheets with the rounded Fe nanoparticles; images at higher magnification (e) show the typical interplanar spacing of MoS_2_ and the FFT in the regions (I), (II), and (III) are reported on the right.

Figure 6a shows as an example a STEM image of the MoS_2_–Fe catalyst and the elemental maps (b-d) obtained by EELS in the same region. The MoS_2_ flake can be clearly identified, and iron is also detected in the same region. Figure 6e shows an RGB image obtained by superimposing the signals of Fe (red), Molybdenum (green), and Sulfur (blue). Figure 7a shows the comparison between Raman spectra acquired on C-felt with MoS_2_ and with MoS_2_ and Fe. The position of the characteristic peak E^1^_2g_, which represents the vibrations parallel to the plane structure, does not change with iron addiction. However, the A_2g_ peak, which is related to the interaction between the layers, through the van der Waals gaps, decreases from 408.3 cm^–1^ to 407.3 cm^–1^. This suggests an interlayer interaction, possibly due to the presence of iron and/or oxygen atoms introduced into the van der Waals gaps. A similar observation has been also reported in ref 61.

**Figure 6:**
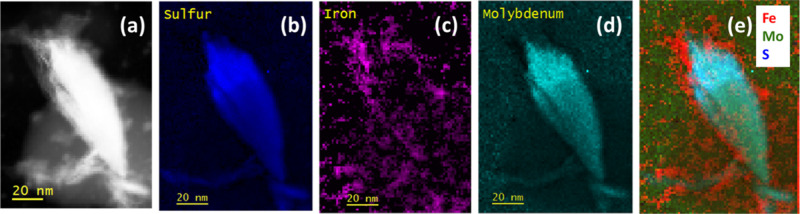
STEM-EELS analyses of C-felt-MoS_2_–Fe: (a) the ADF image of a region of the MoS_2_–Fe catalyst, and the corresponding EELS maps (b–d) of S, Fe, and Mo, respectively, (e) superimposition of the three elements: iron (red), molybdenum (green), and sulfur (blue).

**Figure 7:**
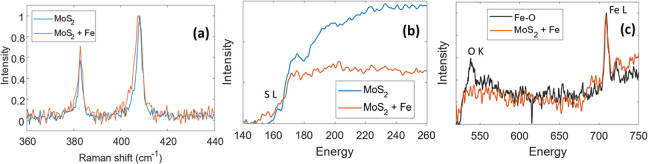
(a) Comparison between Raman spectra of MoS_2_ without and with a Fe catalyst. (b) L_23_ core-level EELS edges of sulfur measured in MoS_2_ and MoS_2_ + Fe. (c) Fe and O core-level EELS edges measured in Fe nanoparticles and MoS_2_ + Fe.

Such an observation is confirmed by the EELS spectra acquired in the region of the core-level edges. Figure 7b shows as blue and red lines the S L_23_ core-level EELS edges measured in MoS_2_ and MoS_2_–Fe, respectively. The edges observed in MoS_2_ alone exhibit two peaks, as typically observed for this material.^62^ However, in the case of addition of iron, a peak at lower energy appears; this can be ascribed to the Fe–S bond, as reported in ref 51. The Fe and O core-level EELS edges are reported in Figure 7c. Spectra of MoS_2_ + Fe shown in Figure 7b,c have been acquired in the same region. In the sample with Fe nanoparticles (black curve in Figure 7c), the signal of the Oxygen K shell at 532 eV is clearly detected because the external shell of the nanoparticles is made of oxides or hydroxides. In contrast, in the MoS_2_–Fe catalyst, only the signal of iron is clearly detected. Therefore, Fe atoms appear to be embedded into the MoS_2_ flake, reasonably bonded to sulfur, since it exhibits a peak at 153 eV. This is consistent with findings that metal nanoparticles often transform into more dispersed states when in contact with MoS_2_, optimizing their catalytic activity (e.g., hydrogen evolution reactions) while maintaining stability under reaction conditions.^63^

The change from isolated Fe-based nanoparticles with a core–shell structure to iron clusters embedded in the MoS_2_ structure can significantly influence the catalyst’s surface properties, affecting its activity, stability, and reaction pathways. The observations are even better evidenced by atomistic simulations.

### 3.2 Atomistic Simulations

To explore the interactions and reactivity of all relevant interfaces in the C-felt-MoS_2_–Fe catalyst, molecular dynamics simulations have been performed for the isolated nanoparticle, the graphite/nanoparticle, and the MoS_2_/nanoparticle systems. Comparing molecular dynamics at different temperatures could provide valuable insights into the reactivity of the two substrates with iron oxide, elucidating the atomistic origin of the catalyst high performance on nitrate electrochemical reduction. To accelerate the exploration of the configurational space and, at the same time, to give enough energy for relevant atomistic transitions, for each system, a single molecular dynamic with an increasing temperature has been performed. MD has been used to sample the configurational space and cross-kinetic barriers, where relevant atomistic transitions are not observable in dynamics under experimental conditions reported in this work. Varying temperature is the key parameter adopted in global structure prediction algorithms like simulated annealing [sim-annealing] and minima hopping [min-hop-1, min-hop-2, min-hop-3]. All molecular dynamics have been integrated with a time step of 1.0 fs. Panels (0) of Figure 8 show the composed structures for C-felt/NP and MoS_2_/NP. Panel (a) of Figure 8 shows the input MD temperature variation during the molecular dynamics. Temperature is increased each 300 ps, allowing a proper equilibration at *T* = 300, 600, 1200, and 1800 K. The last run at a temperature of 1800 K lasts for 500 ps. To properly compare structures and energetics, configurations shown in panels (1–4) of Figure 8 represent the quenched (quenching rate 150 K/ps, stop for temperatures less than 10 K) and relaxed (forces less than 1.0 meV/Å) structures at the end of each temperature. Orange arrows represent the quenching and relaxation procedures, which deliver the structures to be compared. The adopted heating and cooling approach to alter the physical properties of the target system and to sample its potential energy surface represents the basic principle of the well-established simulated annealing algorithms.

**Figure 8:**
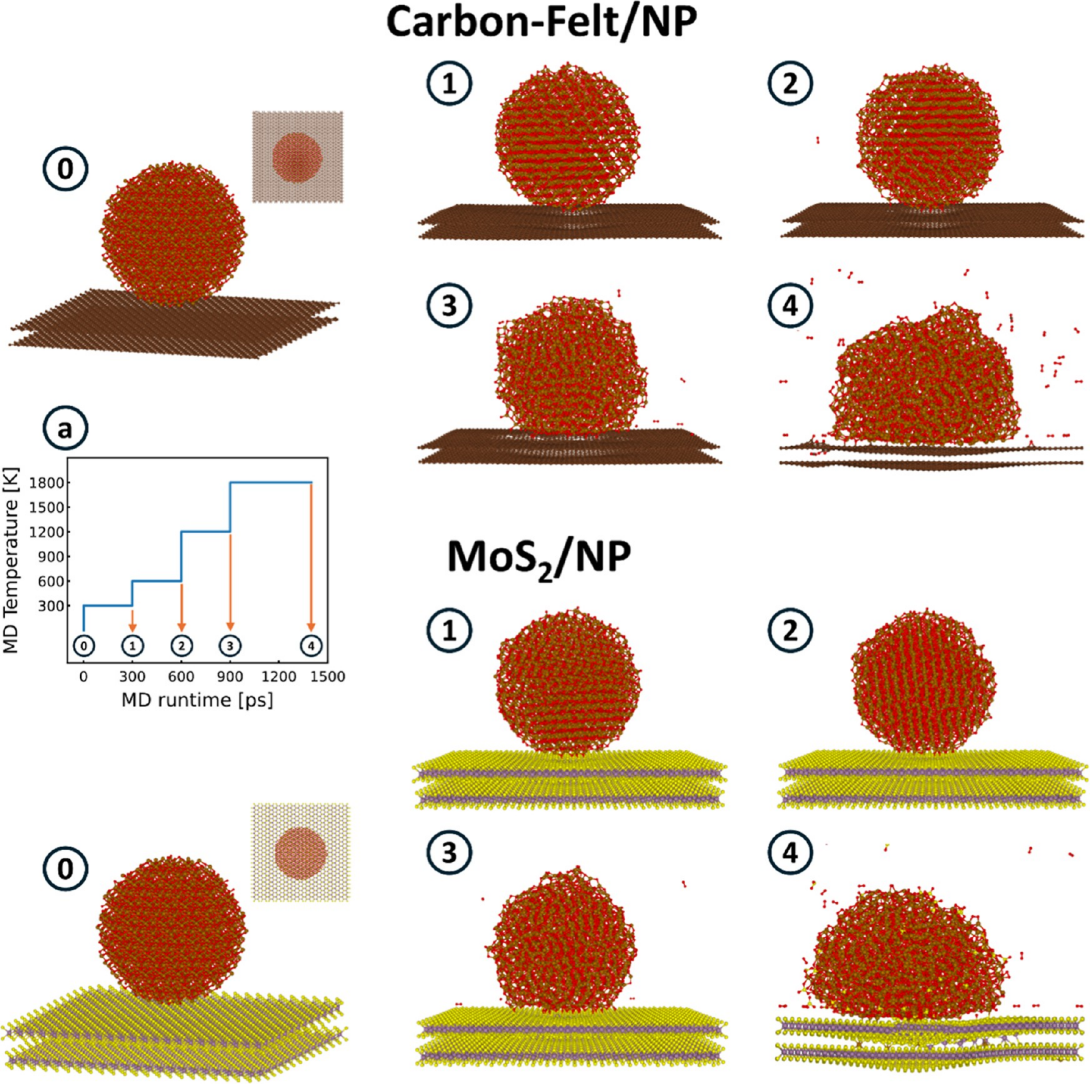
Sampling of various atomistic configurations of the two composed systems made of the Carbon-felt and MoS_2_ interacting with an Fe_2_O_3_ nanoparticle (NP). Panel (0) shows the initial structures, where a Fe_2_O_3_ nanoparticle of 2 nm has been placed on top of a graphite-like Carbon-felt (top structures) or MoS_2_ (bottom structures) substrate. Panel (a) shows the input MD temperature variation during the molecular dynamics. Temperature is increased each 300 ps, allowing a proper equilibration at *T* = 300, 600, 1200, and 1800 K. The last run at a temperature of 1800 K lasts for 500 ps. Configurations shown in panels (1–4) represent the quenched (quenching rate 150 K/ps, stop for temperatures less than 10 K) and relaxed (forces less than 1.0 meV/Å) structures at the end of each temperature. Orange arrows represent the quenching and relaxation procedures. (Oxygen: red; Iron: gold; Carbon: brown; Sulfur: yellow; Molybdenum: purple).

A first inspection of the configurations (1–4) of Figure 8 indicates that iron oxide complexes interact more easily with MoS_2_ than with C-felt. All annealings up to 1800 K do not alter the carbon substrates and the carbon/NP interface. The latter is characterized by few carbon–oxygen bonds, whose number increases with the increasing thermal budget and NP modification. Interlayer diffusion events of oxygen or iron atoms do not take place. The scenario is similar for the MoS_2_/NP system for temperatures up to 1200 K. The most relevant difference is the presence of Fe–S bonds. The MoS_2_/NP interface is characterized by an increasing number of oxygen and iron bonds with S atoms with an increasing temperature. Annealing at 1800 K drastically modifies the MoS_2_/NP system. Relevant diffusion events of iron atoms inter- and intra MoS_2_ layers take place, as well as S diffusion in the iron oxide material. The system starts to be deeply integrated, and iron atoms establish a multitude of connections with sulfur atoms. These configurations well match the characterization results reported in the previous section concerning the A_1g_ peak red shift (see Figure 7a) and the EELS spectra in the region of the core-level edge for the MoS_2_–Fe catalyst (see Figure 7b). Concerning the NP, the increasing temperatures induce the expulsion of oxygen in the form of O_2_ with the reduction of oxygen content in the iron oxide. This is in agreement with the phase diagram of iron oxide at our simulated conditions [FeO-phase diagram], where heating hematite induces a phase transition to magnate and gas O_2_ (hematite contains only Fe^3+^, whereas magnetite contains both Fe^2+^ and Fe^3+^) [FeO-phase-diagram].

Our results agree with the current knowledge which indicate that Iron interacts more easily with sulfur than carbon.^64,65^ Iron has a strong affinity for sulfur due to its soft acid–soft base interaction. This leads to the formation of stable iron–sulfur clusters, sulfides (e.g., FeS, FeS_2_), and other coordination complexes. Iron sulfides (FeS, FeS_2_, Fe_3_S_4_) form naturally and are found in minerals, such as pyrite and troilite. Additionally, Fe–S clusters play crucial roles in biological systems, such as in enzymes and electron transfer proteins. Iron carbides (Fe_3_C, Fe_4_C, etc.) form in steel production, but these are less common in nature and require specific conditions, such as high temperatures. On the other side, Iron forms weaker bonds with carbon in comparison. While Fe–C bonds exist in materials like cementite (Fe_3_C) and organometallic compounds (e.g., ferrocene), they are generally not as strong or prevalent as Fe–S interactions Fe–S bonds generally have lower formation energy, making them easier to form and more stable under standard conditions, while Fe–C bonds require specific conditions (such as high-temperature steelmaking or organometallic synthesis), making them less naturally favorable.

Summarizing the results of the structural characterization, we can conclude that in the MoS_2_–Fe catalyst, iron appears present as single-atom species supported on MoS_2_ nanoflakes forming bonds mainly with S and oxygen.

### 3.3 NO_3_RR Electrochemical Performance

The catalytic performance of the prepared catalysts for the reduction of nitrate to ammonia has been evaluated and compared. Figure 9 shows the Linear Sweep Voltammetry (LSV) curves for the three catalysts tested under two conditions: PBS (phosphate-buffered saline) and PBS with KNO_3_.

**Figure 9:**
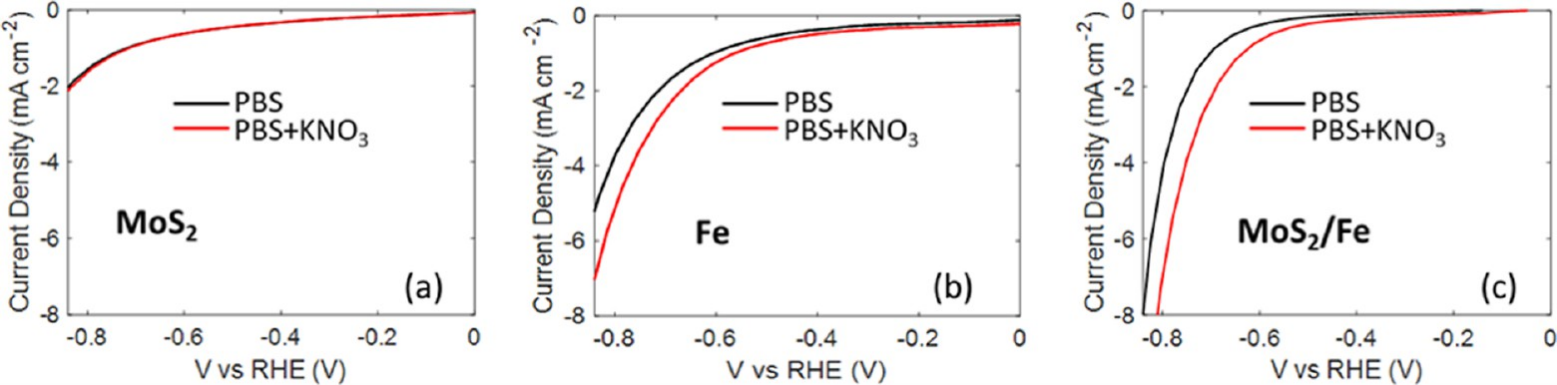
LSV curves of the C-felt electrode in an electrolyte with and without KNO_3_: (a) MoS_2_, (b) Fe, and (c) MoS_2_–Fe catalysts.

The C-felt-MoS_2_ electrode, shown in Figure 9a, is weakly active in the selected potential range in both the electrolytes. Instead, the current density in the electrolyte containing NO_3_^–^ is significantly higher than that without NO_3_^–^, for both C-felt-Fe and C-felt-MoS_2_–Fe catalysts, reported in Figure 9b,c, respectively, suggesting the occurrence of nitrate conversion to ammonia.

In Figure 10, we report the Nyquist plot for the catalysts together with the equivalent circuit adopted for fitting to the data. The electrochemical parameters for the catalysts adopted in our experiments are summarized in Table 1. By fitting data according to the equivalent circuit shown in Figure 10, we can evaluate the resistance of the solution (*R*_s_), the charge transfer resistance (*R*_ct_), related to the reaction kinetics, and the double-layer capacitance (*C*_dL_), linked to the electrochemical surface-active area (ECSA). The resistance of the solution was adopted for the iR correction of the LSV. The C-felt-MoS_2_–Fe catalyst exhibits the lowest *R*_ct_ value of 8.8 Ω, indicating the most efficient charge transfer among the tested materials. This suggests a highly conductive interface and a synergistic interaction between Fe and MoS_2_ that facilitate electron transfer during the nitrate reduction reaction. In contrast, the Fe and MoS_2_ catalysts show significantly higher *R*_ct_ values of 21.8 and 33.2 Ω, respectively, which correlate with their lower catalytic activity. The *C*_dL_ values provide additional confirmation of the enhanced electrochemical exposed active sites of C-felt-MoS_2_–Fe. With a *C*_dL_ of 4.2 mF cm^–2^, Fe–MoS_2_ possesses a larger active surface area compared to MoS_2_ (1.1 mF cm^–2^) but lower than Fe (8.1 mF cm^–2^). This balance suggests that Fe–MoS_2_ benefits from both increased charge transfer efficiency and sufficient active sites to promote nitrate reduction. Moreover, the *C*_dL_ data obtained for the Fe catalyst confirms that the deposition of iron on C-felt led to 3D-nanoparticles with a larger catalytic surface area and consequently more active sites compared to those nucleated on MoS_2_, where the interaction Fe/S resulted in a less rough morphology.

**Figure 10:**
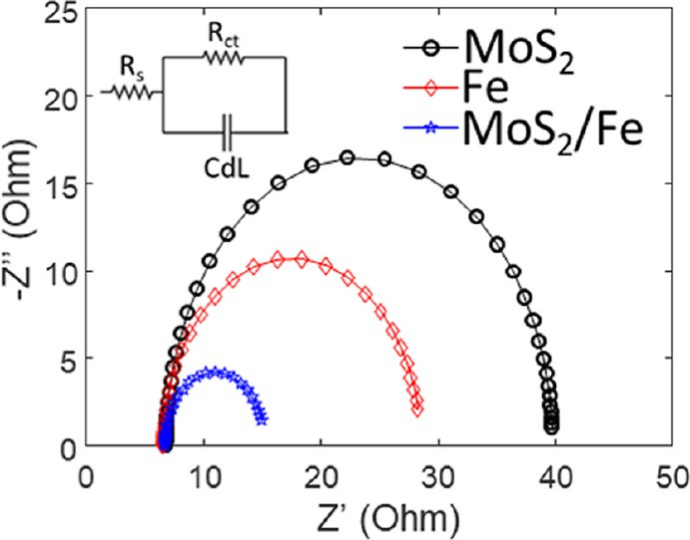
Nyquist plot for the three synthesized catalysts and the equivalent circuit adopted to fit the experimental data.

**Table 1:** Catalysts Electrochemical Parameters Calculated by Fitting the EIS Spectra of Figure 10

sample	*R*_ct_(Ω)	*C*_dL_ (mF cm^–2^)
MoS_2_	33.2	1.1
Fe	21.8	8.1
Fe/MoS_2_	8.8	4.2

The prepared catalysts have been adopted for nitrate reduction to ammonia by chronoamperometry, performed at different voltages for 2400 s. The curves for all the experiments are reported in Figure SI7. After each experiment, the ammonia amount has been evaluated by the indophenol method and for comparison by an ammonia ISE electrode (see Figure SI13). From the measured ammonia moles, the production rate has been evaluated, as plotted in Figure 11a, as a function of the applied potential versus the reversible hydrogen electrode (V vs RHE) for all the studied different catalysts. The Faradaic efficiency (FE) for nitrate conversion to NH_3_, shown in Figure 11b, was evaluated by comparing the NH_3_ moles produced to those expected by the total charge passed through the electrode according to eq 2:

2 $$FE=\frac{8\times F\times {moles}_{NH_{3}}}{Q_{tot}}$$

where F is the Faraday constant (96485C mol^–1^); *Q*_tot_ is the total charge passed through the electrode; and 8 is the number of electrons required to deliver a NH_3_ molecule from a NO_3_^–^ ion.

**Figure 11:**
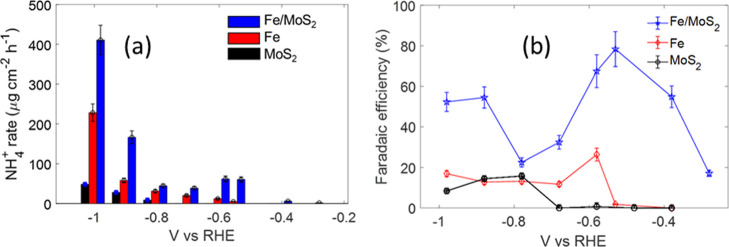
Potential-dependent NH_4_^+^ yield rate (a) and the corresponding Faradaic efficiency evaluated by eq 2 (b).

It is well established from the literature that the conversion of nitrate to ammonia is a complex multi-step reaction, which initially involves the reduction of nitrate to nitrite via a single-electron transfer.^66^ Iron is present in the catalyst in multiple species with oxidation number 0, for metallic Fe in the inner part of the nanoparticles, and +2 or +3 for oxide and/or hydroxides. In addition, we have also found that Fe can be bonded to S within the MoS_2_ structure. All the observed species are reactive in the presence of aqueous solutions with N-based compounds according to the following reactions:^67,68^

3 $${Fe}^{0}+{NO}_{3}^{-}+H_{2}O\to {Fe}^{2+}+{NO}_{2}^{-}+{2OH}^{-}$$

4 $${4Fe}^{0}+{NO}_{3}^{-}+{7H}_{2}O\to {4Fe}^{2+}\;{+NH}_{4}^{+}{+10OH}^{-}$$

5 $${Fe}^{2+}+{2H}_{2}O\to Fe{\left(OH\right)}_{2}+{2H}^{+}$$

6 $${6Fe}^{2+}+{7H}_{2}O+{NO}_{2}^{-}\to {3Fe}_{2}O_{3}+{11H}^{+}+{NH}_{3}$$

All of these reactions may take place. Therefore, in order to get more insight into the occurring reactions at different voltages, we have measured the residual nitrates and the produced nitrites and ammonia after chronoamperometry for 40 min, at different voltages. To this purpose, specific detectors were prepared, as described in refs 43–45 . Measurements were performed in a 0.01 M PBS electrolyte solution (pH = 7). The obtained data were interpolated using calibration curves constructed in PBS (see Materials and Methods). The results are shown in Figure 12.

**Figure 12:**
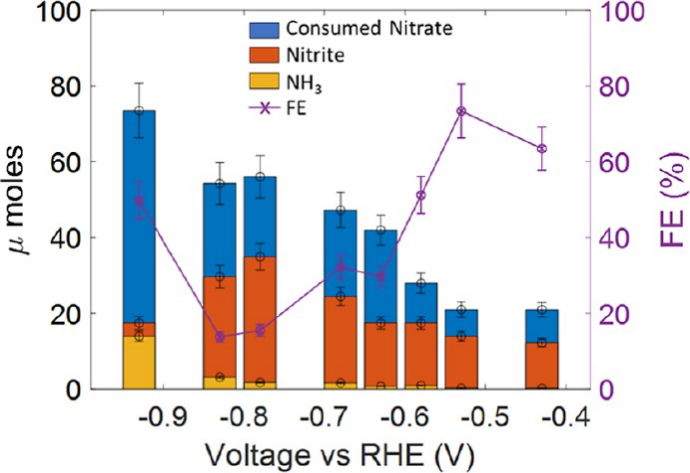
Moles of consumed nitrates, produced nitrites, and ammonia, as a function of voltage during chronoamperometry for 40 min. On the right axis, the Faradaic efficiency for ammonia production is also reported.

The consumption of nitrates increases as the applied voltage becomes more negative. For voltages in the range from −0.4 V to −0.8 V vs RHE, up to 70% of the consumed nitrates is converted into nitrites, while the amount of produced ammonia is low, suggesting that in this voltage range, eq 3 is the dominant reaction. Around −0.8 V vs RHE, the nitrite production has a peak and decreases for more negative values, while the ammonia amount largely increases at voltages lower than −0.9 V vs RHE. It is clear from the nitrite measurements that the depletion in the Faradaic efficiency observed at about −0.8 V vs RHE is due to the higher production of nitrites: electrons are mainly used to convert nitrates in nitrites, instead of producing ammonia. For more negative voltage values, the nitrite amount decreases and the ammonia increases, corresponding to an increase in the Faradaic efficiency. At voltages more negative than −1 V vs RHE, the efficiency again decreases due to the onset of the competing hydrogen evolution reaction.

Overall, MoS_2_ alone does not seem to be a good catalyst for the NO_3_RR while the Fe catalyst exhibits moderate activity, producing ammonia according to eq 3–6, although at lower rate, compared to MoS_2_–Fe. The limited performance of MoS_2_ in this specific reaction could be due to its poor ability to absorb nitrate ions or facilitate the necessary electron transfer reactions for efficient ammonia production. However, when coupled with Fe, its catalytic properties improve significantly, underscoring the need for material combinations that can compensate for individual lacks. The MoS_2_–Fe composite indeed exhibits the highest rate of ammonia production over the tested range, with a maximum of nearly 411 μg  cm^–2^  h^–1^ at −0.98 V vs RHE. Moreover, it demonstrates significantly higher Faradaic efficiency, reaching a peak value of nearly 80% at −0.53 V vs RHE. However, for potential more negative than −0.53 V, the Faradaic efficiency decreases, due to the onset of side reactions, mainly nitrite formation or HER). In contrast, despite iron being involved in the main reactions for nitrate reduction, the Fe catalyst exhibits consistently low Faradaic efficiency, remaining below 20%. This indicates that Fe alone is not an effective catalyst for nitrate electroreduction under these conditions and the combination of iron and molybdenum disulfide produces a positive effect, where each component enhances the overall catalytic activity. The synergy between the two materials results in a catalyst that is more efficient in converting nitrate to ammonia compared to either material used individually.

NH_3_ yield results are consistent with values reported in the literature for similar catalysts (see Table S1) for comparable nitrate concentration values in the electrolyte. It is worth to note that higher production rates and efficiency can be found in literature, and these can be attributed to the use of higher concentrations of electrolytes in the electrochemical cell, which generally enhances the ammonia production rate, as the greater availability of reactant facilitates improved catalytic performance.^69,70^

To assess the recycling stability of the MoS_2_/Fe catalyst, we measured the ammonia moles produced by the same sample at different voltages in the range from −0.28 V to −0.98 V vs RHE, with a step of 0.1 V (Experiments 1 and 2) for a total of 12 h of operation. We also performed stress test on the same sample (Experiment 3) by fixing the applied voltage (*V*_RHE_ = −0.98 V) and varying the time (20, 40, and 60 min). Results are summarized in Figures SI8 and SI9 of the Supporting Information. The data obtained at the same voltage are in excellent agreement with each other and allow us to state that the Fe–MoS_2_ catalyst we developed is stable, and results are reproducible. This is confirmed by structural analyses, performed by SEM after conducting Experiments 1, 2, and 3. The catalyst before and after usage for 16 h exhibits the same morphology, as shown in the comparison of Figure SI10. EDS analyses acquired for the fresh and used catalyst, shown in Figures SI11 and SI12, respectively, both indicate the presence of iron-based nanoparticles and MoS_2_ flakes, in which Fe atoms appear embedded to, reasonably bonded to sulfur.

## 4 Conclusions

In conclusion, our study demonstrates that the Fe–MoS_2_ catalyst, deposited on carbon felt, offers a promising and sustainable approach for the electrochemical reduction of nitrate to ammonia in a range of nitrate concentrations (500 ppm) very close to that typical of wastewater. By leveraging a simple and low-cost fabrication process that combines liquid-phase exfoliation, electrophoretic deposition, and a solution-based iron drop-casting method, we have achieved a composite catalyst with superior performance compared to its individual components. The synergistic interaction between iron and MoS_2_ not only enhances the catalytic activity—yielding nearly 411 μg  cm^–2^  h^–1^ of ammonia at −0.98 V vs RHE and reaching a peak Faradaic efficiency of approximately 80% at – 0.53 V vs RHE—but also ensures efficient nitrate reduction while minimizing competing reactions such as hydrogen evolution. These features, coupled with the use of low-cost materials and scalable preparation techniques, underline the potential of the Fe–MoS_2_ nanosheets as efficient, earth-abundant catalysts for ammonia electrosynthesis from nitrate, offering a sustainable strategy for wastewater valorization and green ammonia production.

## Supplementary Material



## Notes

Additional description of experimental setup experimental details and methods including micrographs and compositional analyses; additional electrochemical measurements; validation of results by additional methods; and comparison with data and catalysts already present in the literature (PDF)
