# Supplementary material for: High-Performance Fe–MoS2 Electrocatalyst for Efficient Nitrate Reduction to Ammonia: Synergistic Design for Sustainable Ammonia Production
Source: ACS Sustain Chem Eng. 2025 Oct 22;13(43):18472–85. doi: 10.1021/acssuschemeng.5c04274 (PMC12588300; doi:10.1021/acssuschemeng.5c04274)
Supplement: Supplementary file 1 [file sc5c04274_si_001.pdf]

SUPPLEMENTARY INFO FOR:

High Performance Fe-MoS$_2$ Electrocatalyst for Efficient Nitrate Reduction to Ammonia: Synergistic Design for Sustainable Ammonia Production

N. Marino† (*), R. G. Milazzo† (*), R. Farina †,‡, C. Bongiorno†, G. Fisicaro†, S. Libertino†, S. A. Lombardo†, S. M. S. Privitera† (*)

† Institute for Microelectronic and Microsystems (IMM), National Research Council (CNR), Zona Industriale VIII Strada 5, 95121 Catania, Italy

‡ Department of Chemical Sciences, University of Catania, Viale Andrea Doria, 6, 95125 Catania, Italy

Corresponding authors:
gabriella.milazzo@imm.cnr.it
nino.marino@cnr.it
stefania.privitera@cnr.it

Number of pages S1-S12

List of Figures:

Figure SI 1: A schematic of the 3-electrodes electrochemical cell adopted in the experiments, it's a typical 2-compartments configuration, the anode and the cathode are set apart by a ZIRFON® separator.

Figure SI 2: Indophenol method for ammonia quantification: (a) absorbance acquired solutions with different concentrations and (b) the corresponding calibration curve at 660nm.

Figure Si 3: Calibration curves (blue dots and dashed red line) for NO3- concentration in 0.01 M PBS electrolyte solution (pH 7) in a range from 0.25 mM to 9 mM.

Figure SI 4: Calibration curves for NO2- concentration in 0.01 M PBS electrolyte solution (pH 7) in a range from 0.05 mM to 6 mM.

Figure SI 5: (a) and (b) SEM micrographs at different magnification of C-felt with electrodeposited MoS2 composed of agglomerated nanoparticles, each approximately 30 nm in size as shown in the inset; (c) EDX maps acquired in the area enclosed by the yellow rectangle with the corresponding spectrum showing the presence of Mo Lα and S Kα lines, superimposed at 2.33keV.

Figure SI 6: (a) SEM micrograph of C-felt with MoS2 and Fe; (b) EDX spectrum and maps acquired in the area enclosed by the red rectangle with the corresponding spectrum showing the presence of Fe, O, Mo and S

signals, the molybdenum signal is typically overshadowed by that of iron deposited on its surface, but becomes distinguishable when molybdenum is present alone on the substrate.

Figure SI 7: Chronoamperometry measurements at different reduction potentials acquired for (a) C-felt MoS2, (b) C-felt Fe and (c) C-felt MoS2/Fe.

Figure SI 8: Recycling stability for MoS2/Fe catalyst showing the produced NH3 moles after stress measurements of 40min at different applied voltages.

Figure SI 9: Stability test on MoS2/Fe catalyst obtained for different stress times but with the same applied voltage V=-0.98V vs RHE.

Figure SI10: SEM micrograph of the catalyst as prepared (a), and after usage for 16 hours (b).

Figure SI11: SEM images at high magnification and Elemental maps obtained by EDX in the fresh Fe-MoS2 catalyst.

Figure SI12: SEM image and elemental maps obtained by EDX for the catalyst used for 16 hours.

Figure SI13: Validation of ammonia production by ammonia ISE, (a) electrode calibration curve and (b) comparison of values measured by indophenol and ISE methods respectively.

List of Tables

Table S1: Comparison of the catalytic performances of iron with other reported NO3–RR electrocatalysts under ambient conditions.

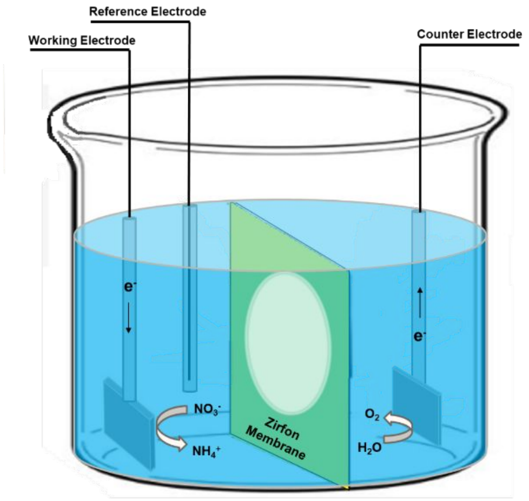

Figure SI 1: A schematic of the 3-electrodes electrochemical cell adopted in the experiments, it's a typical 2-compartments configuration, the anode and the cathode are set apart by a ZIRFON® separator.

Modified indophenol method for ammonia quantification

For ammonia quantification 1 ml of the electrolyte was extracted from the reaction cell and added with 1 ml of a 1 M NaOH solution containing salicylic acid and sodium citrate. Subsequently, 0.5 ml of 0.05 M NaClO and 0.3 ml of 1 wt% (sodium nitroferric cyanide) were introduced into the solution. Following a 2-hour incubation period at room temperature and in the dark, the ultraviolet–visible absorption spectrum was recorded, and the concentration of NH_3 was determined based on the absorbance at a wavelength of 660

nm. The calibration curve was built up by using standard ammonia solutions. UV-Vis spectra of solutions with known concentration as shown in Fig. SI2 (a). The corresponding calibration curve is plotted in Fig. SI2 (b).

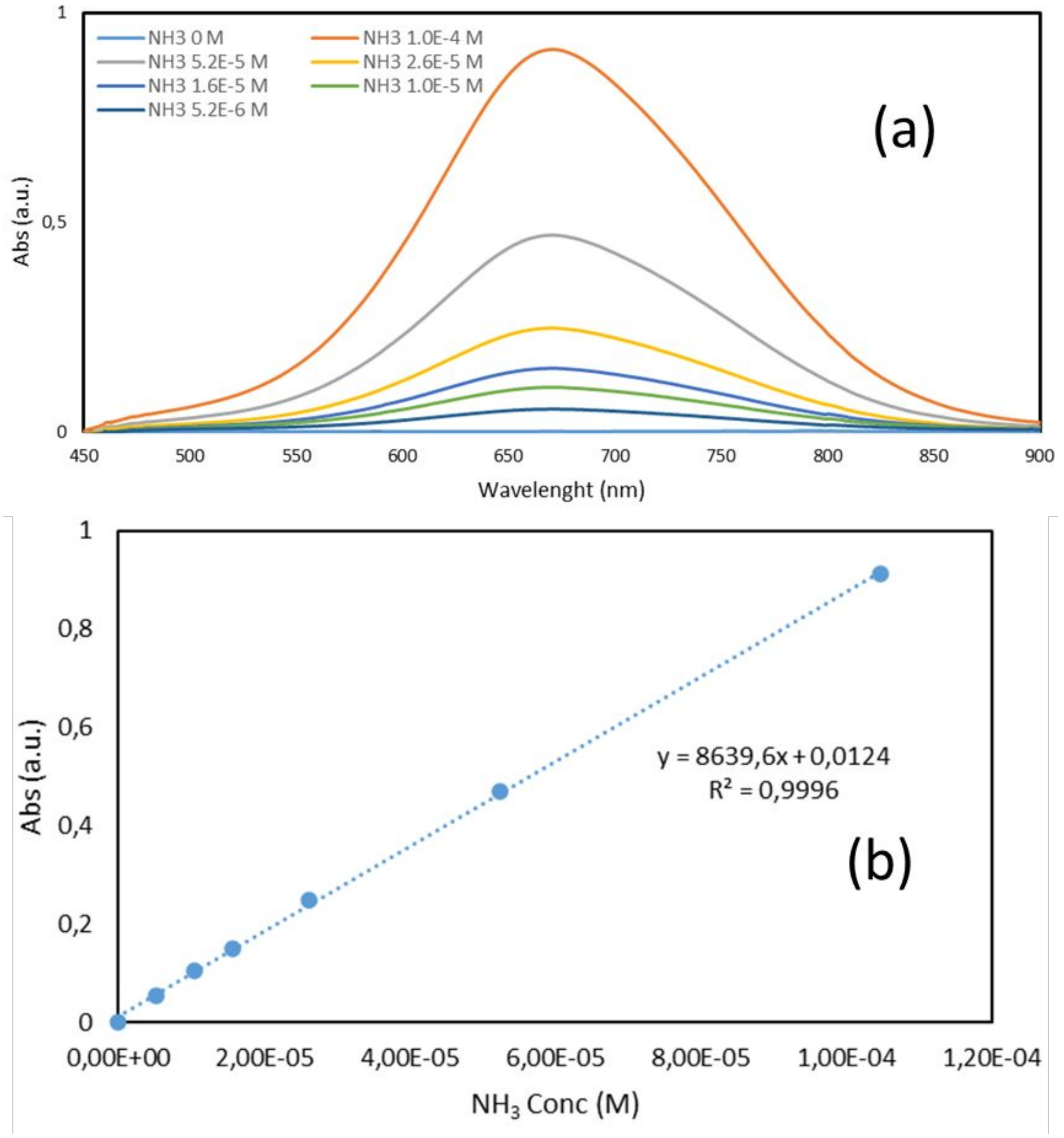

Figure SI 2: Indophenol method for ammonia quantification: (a) absorbance acquired solutions with different concentrations and (b) the corresponding calibration curve at 660nm.

Nitrates and Nitrites quantification

Nitrate (NO_3^-) and nitrite (NO_2^-) concentrations in the solutions, before and after the chronoamperometric experiments, were quantified using innovative electrochemical sensors based on screen-printed electrodes (SPEs). The NO_3^- sensor was fabricated by electrodepositing copper (Cu) microflowers onto the carbon working electrode of the SPE via cyclic voltammetry, using a 0.1 M KCl electrolyte. The device was calibrated

in 0.01 M phosphate buffer solution (PBS, pH 7) from 0.25 mM to 9 mM. The PBS concentration used is the same used for chronoamperometric ammonia production; thus, calibration and measurements are performed on the same solution. The corresponding calibration curve, exhibiting a linear dynamic range, is shown in Figure SI 3. Based on the experimental data collected before and after the chronoamperometric process, a decrease in nitrate concentration was observed, from an initial value of 6 mM to 4.6 mM (red stars in the figure), after ammonia production. The data are also shown in the figure.

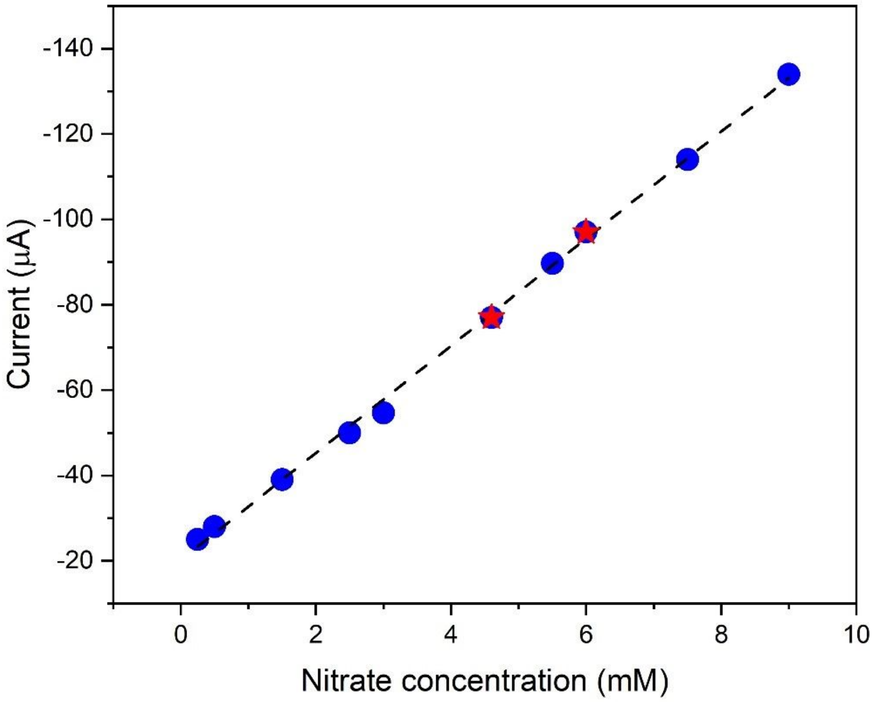

Figure Si 3 Calibration curves (blue dots and dashed red line) for NO3- concentration in 0.01 M PBS electrolyte solution (pH 7) in a range from 0.25 mM to 9 mM. A linear trend is observed. Experimental points before and after ammonia production indicate a initial value of 6 mM and final value of 4.6 mM (red stars).

The NO_2^- sensor was fabricated by co-electrodeposition of copper (Cu) and manganese (Mn) on a porous carbon working electrode, also via cyclic voltammetry in KCl. Sensor calibration curve was performed under the same PBS conditions reported above for the nitrate sensor, in a range from 0.05 mM to 6 mM. Also in this case, a linear dynamic range is observed. As shown in Figure SI 4, experimental data revealed an increase in nitrite concentration from 0 mM to 0.8 mM (red star in the figure) after the chronoamperometric ammonia production experiment.

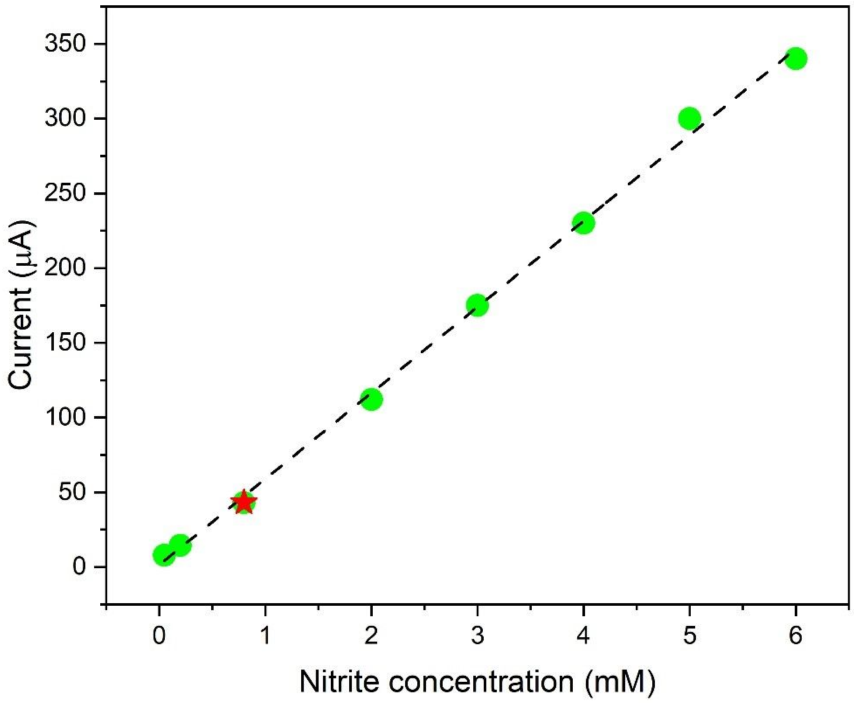

Figure SI 4: Calibration curves for NO2- concentration in 0.01 M PBS electrolyte solution (pH 7) in a range from 0.05 mM to 6 mM. A linear trend is observed. Experimental points before and after ammonia production indicate an initial value of 0 mM and final value of 0.8 mM (red star).

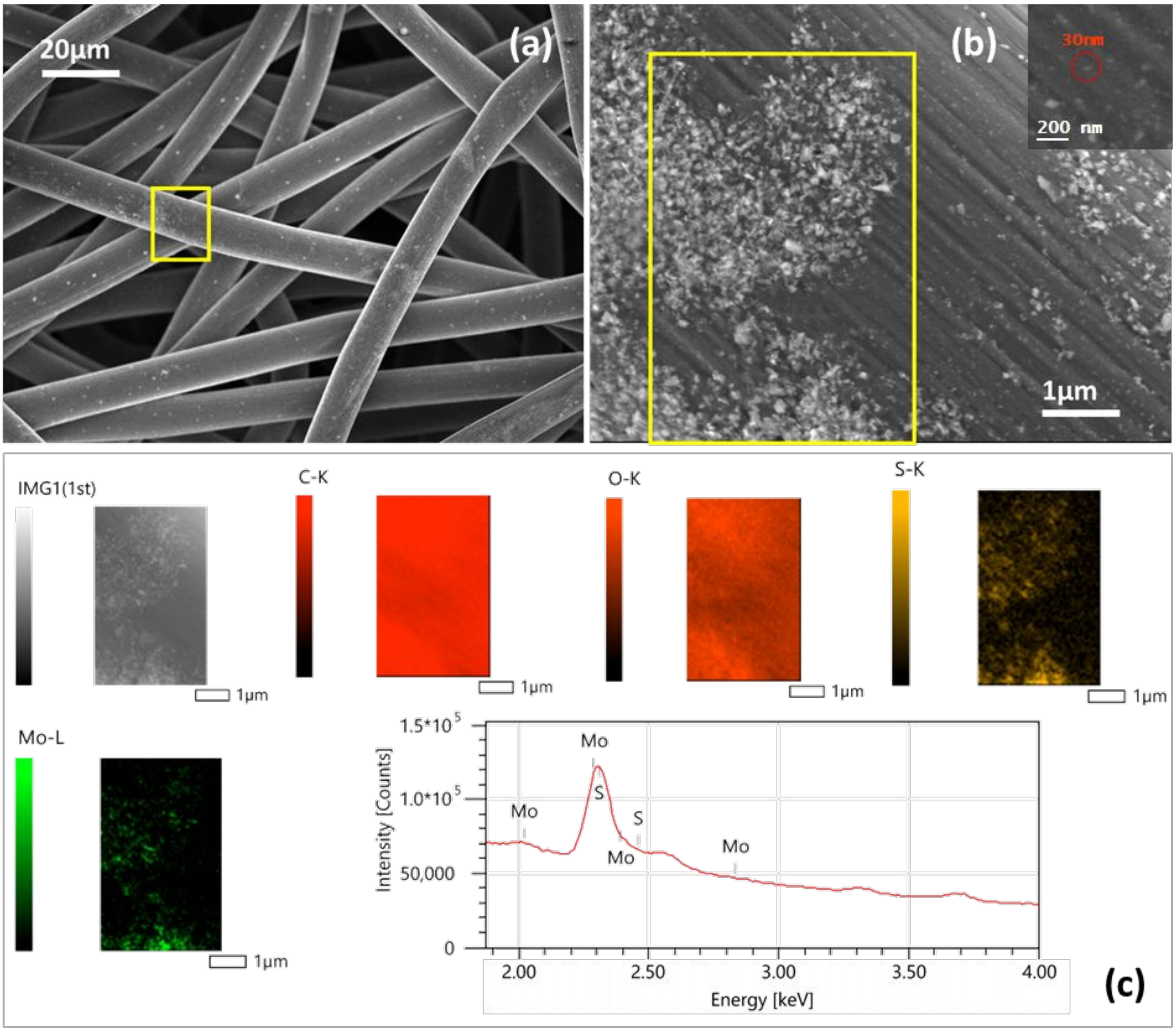

Figure SI 5: (a) and (b) SEM micrographs at different magnification of C-felt with electrodeposited MoS2 composed of agglomerated nanoparticles, each approximately 30 nm in size as shown in the inset; (c) EDX maps acquired in the area enclosed by the yellow rectangle with the corresponding spectrum showing the presence of Mo Lα and S Kα lines, superimposed at 2.33keV.

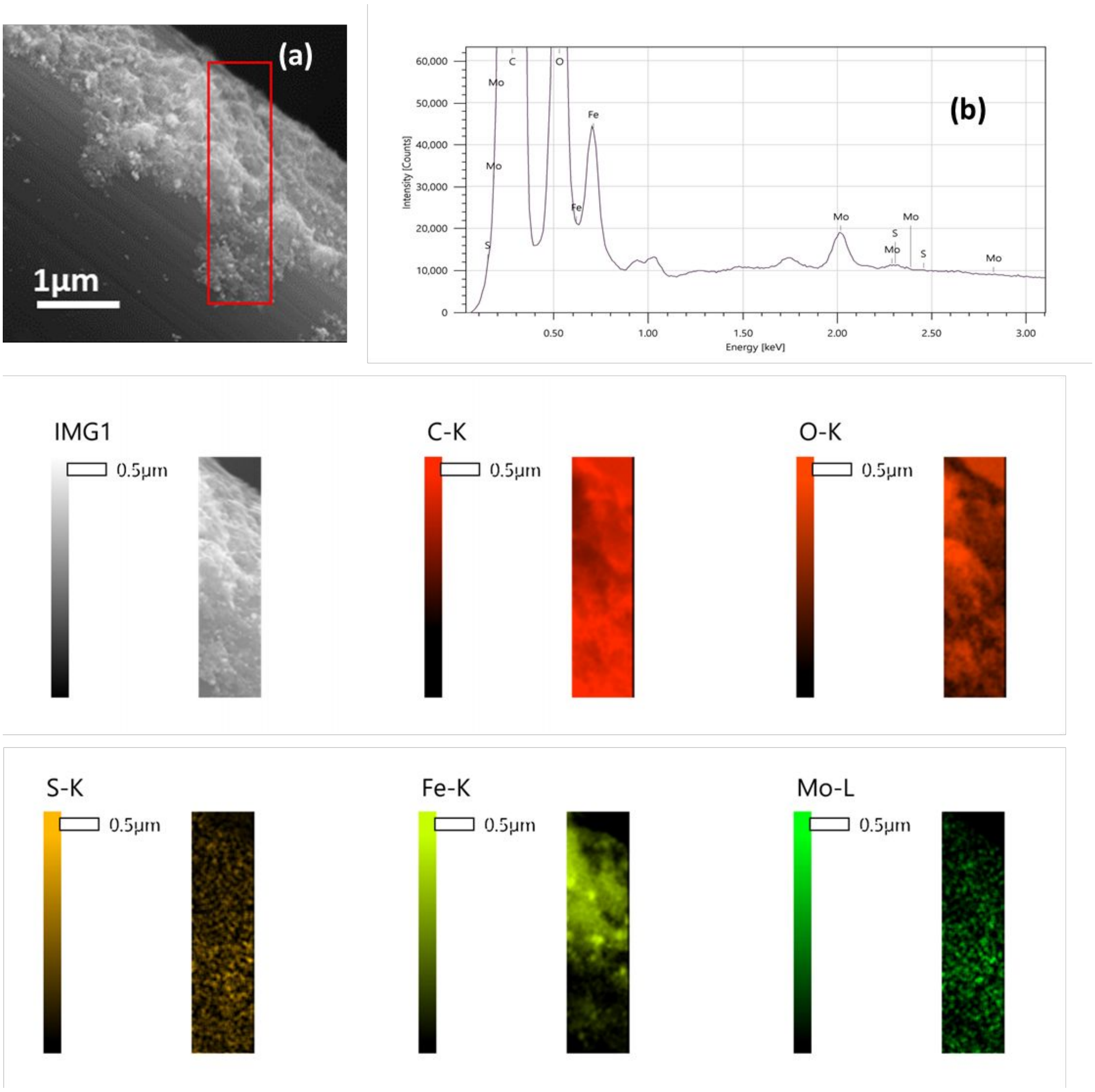

Figure SI 6 (a) SEM micrograph of C-felt with MoS2 and Fe; (b) EDX spectrum and maps acquired in the area enclosed by the red rectangle with the corresponding spectrum showing the presence of Fe, O, Mo and S signals, the molybdenum signal is typically overshadowed by that of iron deposited on its surface, but becomes distinguishable when molybdenum is present alone on the substrate.

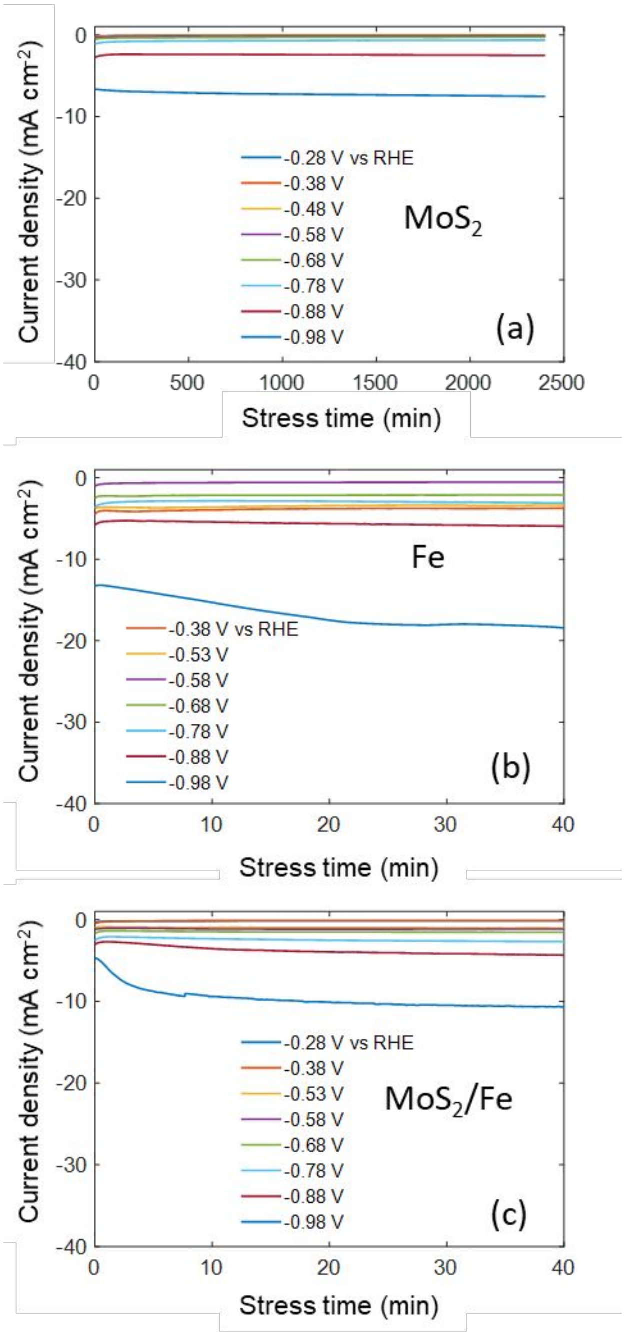

Figure SI 7: Chronoamperometry measurements at different reduction potentials acquired for (a) C-felt MoS2, (b) C-felt Fe and (c) C-felt MoS2/Fe.

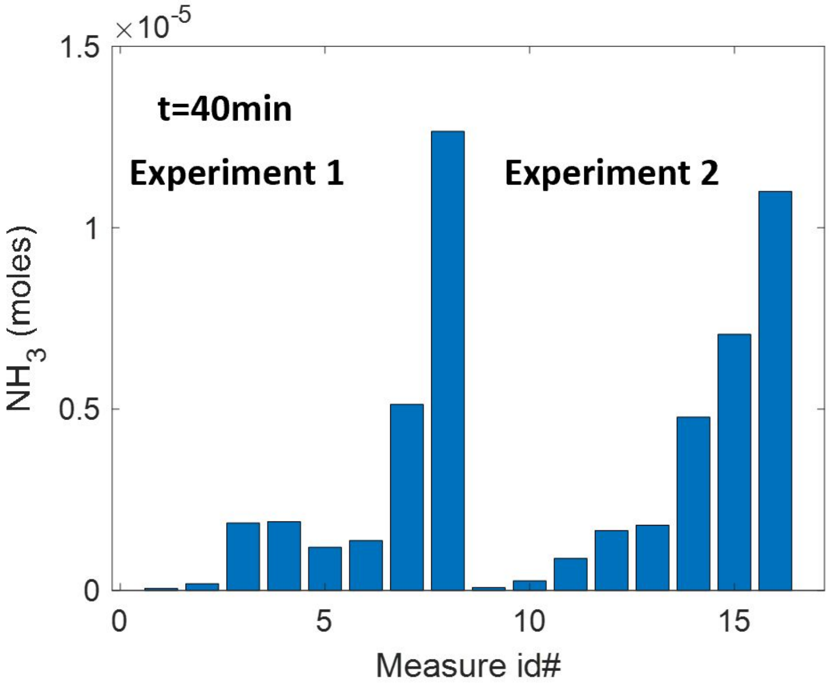

Figure SI 8: Recycling stability for MoS2/Fe catalyst showing the produced NH3 moles after stress measurements of 40min at different applied voltages. We adopted the same sample for two distinct complete experiments (labelled as experiment 1 and experiment 2).

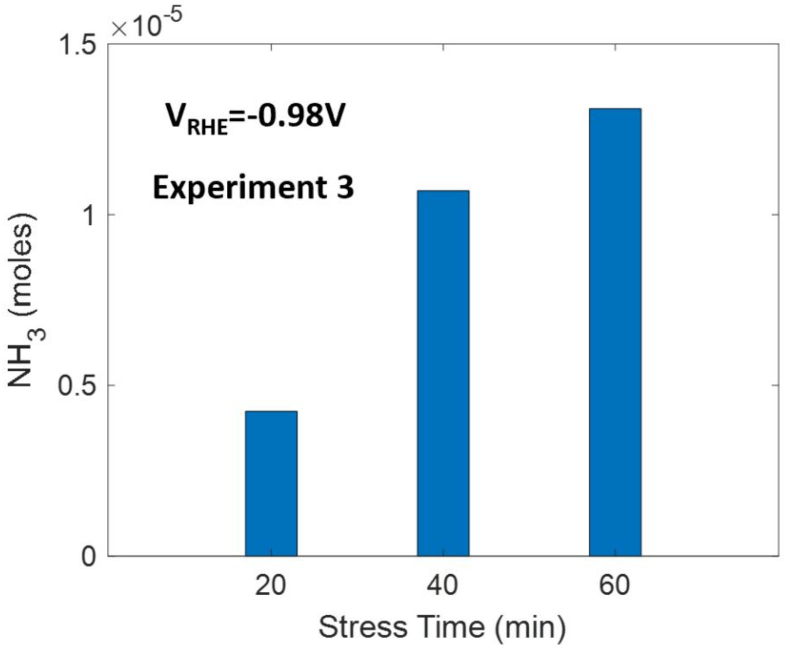

Figure SI 9: Stability test on MoS2/Fe catalyst obtained for different stress times but with the same applied voltage V=-0.98V vs RHE. Measurements are repeated with the same sample used for Experiment 1 and 2.

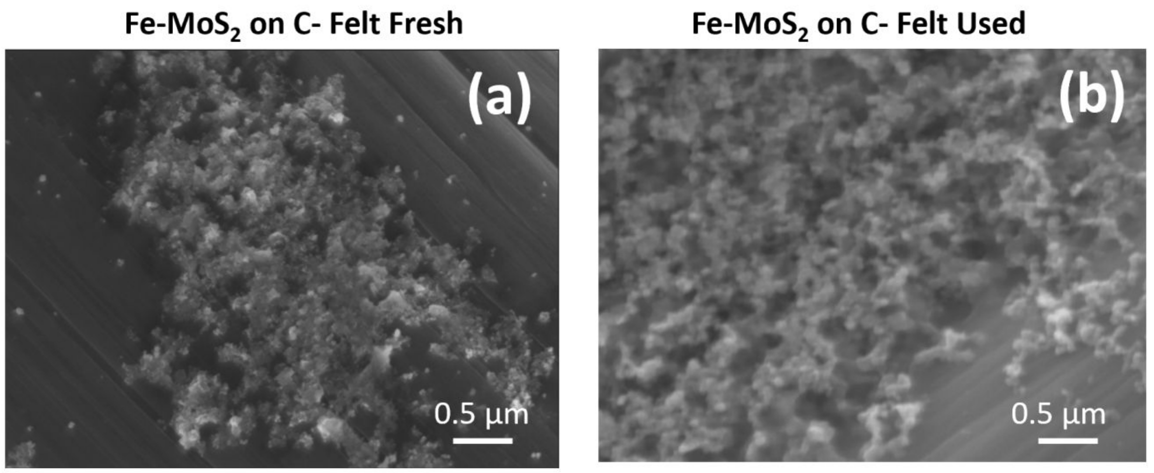

Figure SI10: SEM micrograph of the catalyst as prepared (a), and after usage for 16 hours (b).

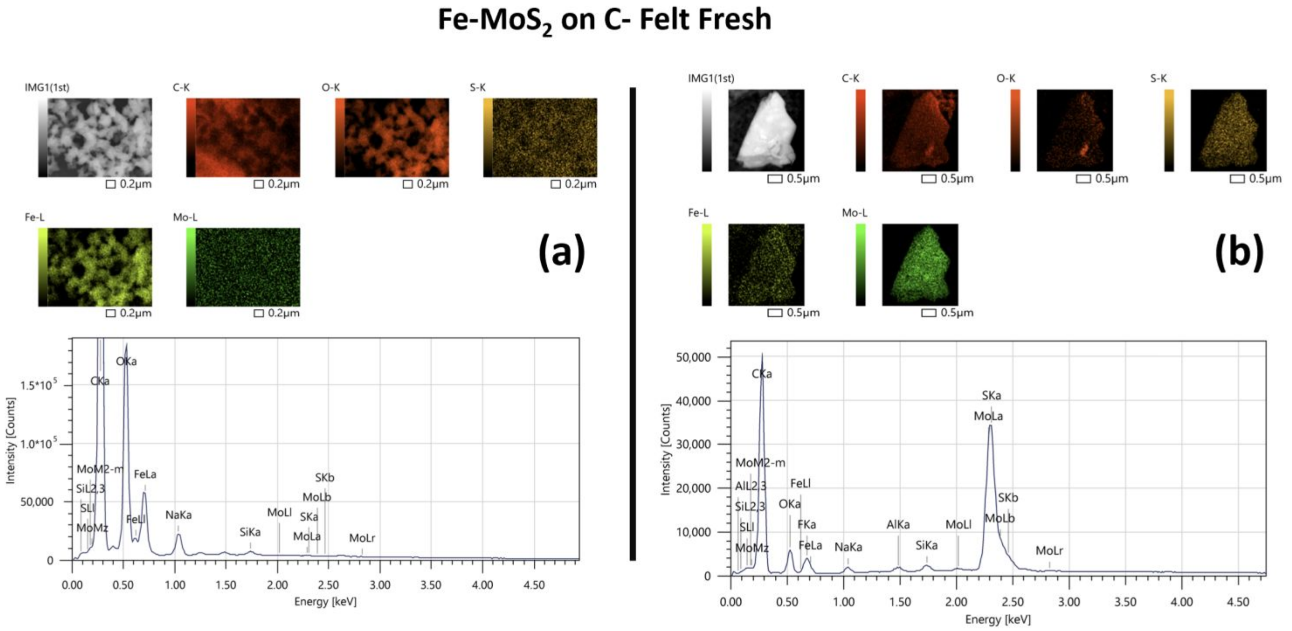

Figure SI11: SEM images at high magnification and Elemental maps obtained by EDX in the fresh Fe-MoS2 catalyst.

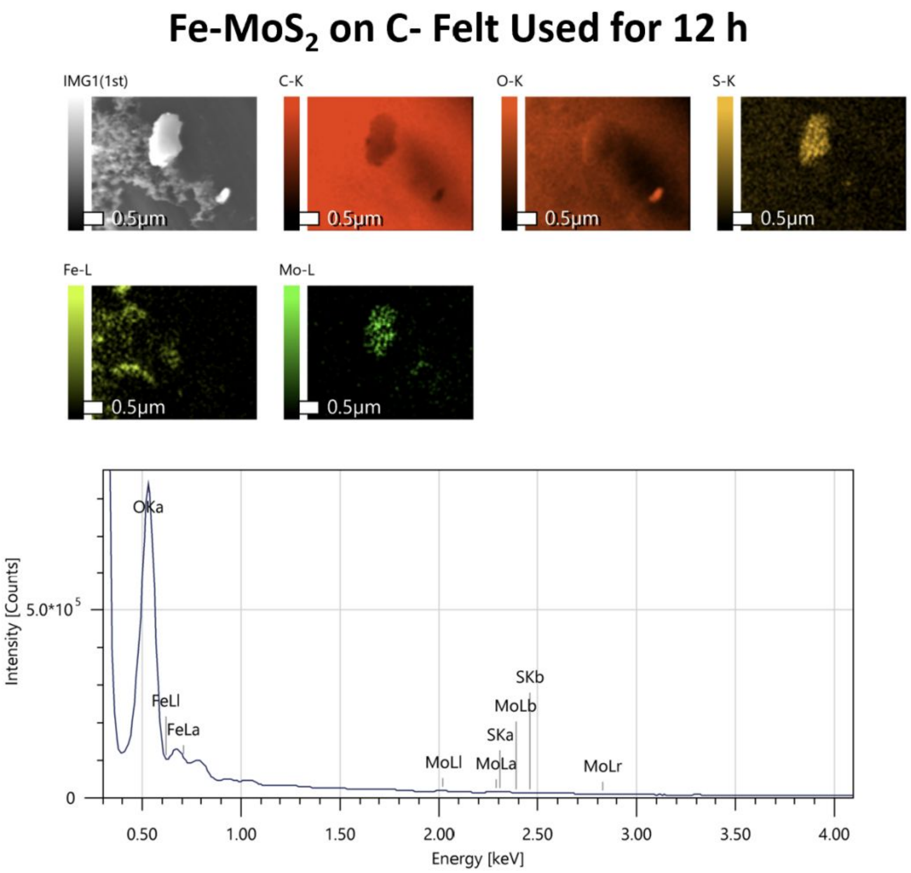

Figure SI12: SEM image and elemental maps obtained by EDX for the catalyst used for 16 hours.

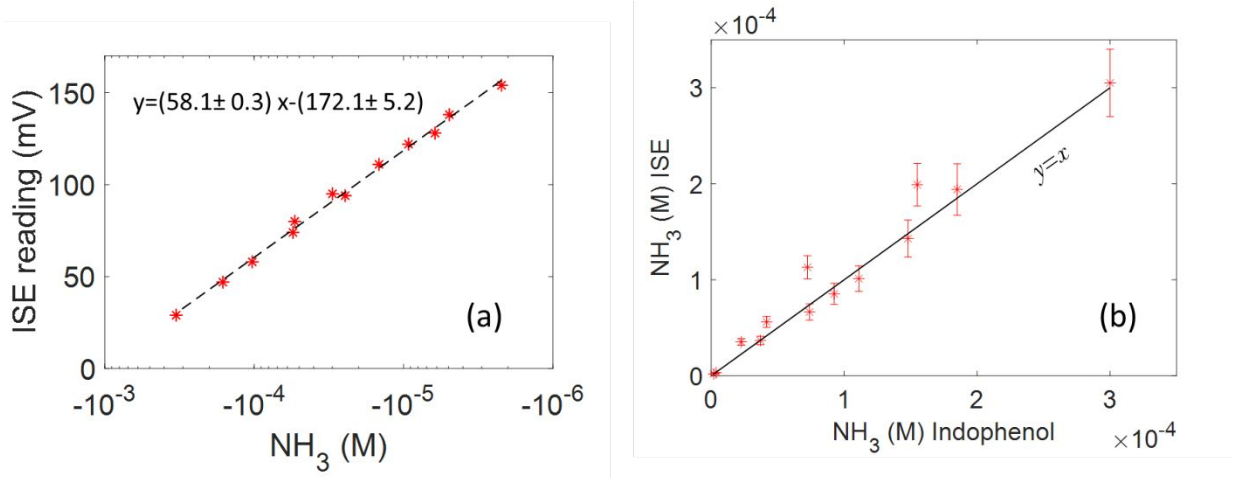

Figure SI13: Validation of ammonia production by ammonia ISE, (a) electrode calibration curve and (b) comparison of values measured by indophenol and ISE methods respectively.

Catalyst	Experimental	NH_3 yield rate (µg cm^{-2} h^{-1})	NH_3 Yield rate (µg mg_{cat}^{-1} h^{-1})	FE(%)	Ref
C-felt/MoS_2/Fe	0.01M PBS + 5mM NO_3^-	411		80%	This work
S vac MoS_2	0.1M Na_2SO_4 + 5 mM NO_3^- -0.6V	333.9 (3mg catalyst weight)	111.3	78.04%	[1]
CC/MoS_2/Fe	0.1 M of Na_2SO_4 + 100mM NaOH 0.1M	510		~98 ± 8.8 % (-0.50V vs.RHE)	[2]
Fe_3O_4 /SS	0.1 M NaOH + 0.1 M $NaNO_3$	10145		91.5% (-0.5V)	[3]
CC/MoS_2/Fe	1 M LiCl + 0.25 M $LiNO_3$	9750		90%	[4]
(Fe_2O_3 NRs/CC)	0.5 M Na_2SO_4 + 0.1 M $NaNO_3$	5578		69.76%	[5]
Fe SAC	0.1M K_2SO_4 and 0.5M KNO_3	7820	20000	75%	[6]

Table S1: Comparison of the catalytic performances of iron with other reported NO_3^-RR electrocatalysts under ambient conditions.

[1] J. Wang, Z. Sun, Y. Li, L. Guo, Y. Wang, C. Fan, Y. Wang, R. Li, X. Zhang, F. Li, Z. Yu, J. Liu Sulfur vacancy MoS2 for electrocatalytic reduction of nitrate to ammonia with enhanced selectivity, Journal of Alloys and Compound, **2023**, 955, 170199;

[2] J. Li, Y. Zhang, C. Liu, L. Zheng, E. Petit, K. Qi, Y. Zhang, H. Wu, W. Wang, A. Tiberj, X. Wang, M. Chhowalla, L. Lajaunie, R. Yu, D. Voiry *Adv. Funct. Mater.* **2022**, *32*, 2108316.

[3] X. Fan, L. Xie, J. Liang, Y. Ren, L. Zhang, L. Yue, T. Li, Y. Luo, N. Li, B. Tang, Y. Liu, S. Gao, A. A. Alshehri, Q. Liu, Q. Kong, X. Sun IN situ grown Fe3 O4 particle on stainless steel: a highly efficient electrocatalyst for nitrate reduction to ammonia Nano Research **2022** 15(4), 3050-3055;

[4] J. Ding, X. Hou, Y. Qiu, S. Zhang, Q. Liu, J. Luo, X. Liu. *Inorganic Chemistry Communications*, **2023**, 151, 110621.

[5] T. Li, C. Tang, H. Guo, H. Wu, C. Duan, H. Wang, F. Zhang, Y. Cao, G. Yang, Y. Zhou ACS Appl. Mater. Interfaces **2022**, 14, 44, 49765-49773.

[6] Z.Y. Wu, M. Karamad, X. Yong, Q. Huang, D.A. Cullen, P. Zhu, et al., *Nat. Commun,* **2021**, 12, 2870.
